# Aberrant NOVA1 function disrupts alternative splicing in early stages of amyotrophic lateral sclerosis

**DOI:** 10.1007/s00401-022-02450-3

**Published:** 2022-07-01

**Authors:** Florian Krach, Emily C. Wheeler, Martin Regensburger, Tom Boerstler, Holger Wend, Anthony Q. Vu, Ruth Wang, Stephanie Reischl, Karsten Boldt, Ranjan Batra, Stefan Aigner, John Ravits, Juergen Winkler, Gene W. Yeo, Beate Winner

**Affiliations:** 1 Department of Stem Cell Biology, University Hospital Erlangen, Friedrich-Alexander University of Erlangen-Nürnberg (FAU), Erlangen, Germany; 2 Department of Cellular and Molecular Medicine, University of California San Diego, La Jolla, CA USA; 3 Center of Rare Diseases Erlangen (ZSEER), University Hospital Erlangen, Friedrich-Alexander University of Erlangen-Nürnberg (FAU), Erlangen, Germany; 4 Department of Molecular Neurology, University Hospital Erlangen, Friedrich-Alexander University of Erlangen-Nürnberg (FAU), Erlangen, Germany; 5 Department of Neurosciences, University of California San Diego, La Jolla, CA USA; 6 Core Facility for Medical Bioanalytics, Institute for Ophthalmic Research, University of Tübingen, Tübingen, Germany

## Abstract

Amyotrophic lateral sclerosis (ALS) is a fatal disease characterized by aberrant alternative splicing (AS). Nuclear loss and cytoplasmic accumulation of the splicing factor TDP-43 in motor neurons (MN) are hallmarks of ALS at late stages of the disease. However, it is unknown if altered AS is present before TDP-43 pathology occurs. Here, we investigate altered AS and its origins in early stages of ALS using human induced pluripotent stem cell-derived motor neurons (MNs) from sporadic and familial ALS patients. We find high levels of the RNA-binding proteins NOVA1, NOVA2, and RBFOX2 in the insoluble protein fractions and observe that AS events in ALS-associated MNs are enriched for binding sites of these proteins. Our study points to an early disrupted function of NOVA1 that drives AS changes in a complex fashion, including events caused by a consistent loss of NOVA1 function. NOVA1 exhibits increased cytoplasmic protein levels in early stage MNs without TDP-43 pathology in ALS *postmortem* tissue. As nuclear TDP-43 protein level depletes, NOVA1 is reduced. Potential indications for a reduction of NOVA1 also came from mice over-expressing TDP-43 lacking its nuclear localization signal and iPSC-MN stressed with puromycin. This study highlights that additional RBP-RNA perturbations in ALS occur in parallel to TDP-43.

**Supplementary Information:**

The online version contains supplementary material available at 10.1007/s00401-022-02450-3.

## Introduction

Amyotrophic lateral sclerosis (ALS) is a rare, fatal neurodegenerative disease that progressively affects motor neurons (MNs) in the motor cortex, brainstem, and spinal cord [30]. The majority of ALS cases occur sporadically (sALS) and less than 10% are monogenic [30]. Nuclear loss, pathological cytoplasmic aggregation [4, 48], and hyperphosphorylation of the RNA-binding protein (RBP) TDP-43 [47] are frequently present in spinal and cortical MNs in ALS patients at late stages of the disease. Underscoring its central role, pathogenic variants in the TARDBP gene that encodes for TDP-43, have also been discovered in ALS patients [61].

A prevailing hypothesis is that TDP-43 nuclear loss-of-function and a toxic cytoplasmic gain-of-function of TDP-43 in insoluble aggregates contribute to the neuronal vulnerability in TDP-43 proteinopathies [11, 19, 23, 38, 69]. Early studies, modeling the loss-of-function, utilized transcriptome-wide cross-linking and immunoprecipitation (CLIP) and RNA-seq and identified that TDP-43 mediates RNA splicing primarily by interacting with UG-rich intronic sequences [52, 64]. Interestingly, aberrant alternative splicing (AS) is frequently observed in *postmortem* tissue of ALS patients [12, 54]. Later studies further highlighted a subset of unannotated human exons (cryptic) normally repressed by TDP-43 in the nucleus [37]. Recently, inclusion of a human-specific cryptic exon in the transcript of the microtubule regulator Stathmin2 (STMN2) has been identified upon TDP-43 depletion, resulting in the generation of a nonsense mediated decay mRNA isoform [31, 46]. These studies also show that this isoform of STMN2 is present in sALS patient samples [31, 46].

Transient, non-lethal stress in induced pluripotent stem cell-derived motor neurons (iPSC-MN) was previously shown to induce the formation of cytoplasmic TDP-43 aggregates [21], leading to signs of aberrant TDP-43 function such as inclusion of the TDP-43-associated STMN2 cryptic exon [41]. In our previous work in patient iPSC-MN with pathogenic variants in hnRNP A2/B1 causing severe neurodegeneration, we found that chemical stress was required to induce hnRNP A2/B1 to move into the cytoplasm [44]. However, even in the absence of stress, abnormal AS changes in patient iPSC-MN were observed suggesting that aberrant AS is an early sign of disease while cytoplasmic localization of RBPs may be a secondary, end-stage feature [44].

These studies collectively suggest that perturbation of RBP-splicing networks is a key component of ALS and may be an early event preceding cytoplasmic localization and aggregation of abnormal RBP-RNA complexes. Here, we investigated the origins of altered AS in early stages of sporadic and familial ALS and discovered new aberrant RBP-splicing networks. We utilized mass spectrometry to identify RBPs that exhibit increased insolubility in iPSC-MNs from ALS patients compared to controls. Focusing on splicing factors NOVA1, NOVA2, and RBFOX2, we applied enhanced CLIP methodology and discovered an enriched frequency of RNA binding of these proteins proximal to ALS-associated AS events. Of these, deeper evaluation of NOVA1 revealed elevated protein levels in the cytoplasm of ALS MNs without TDP-43 pathology in *postmortem* tissue. Exogenous expression and genetic knock out of NOVA1 revealed a complex disruption of NOVA1 function, including events caused by a consistent loss of NOVA1 function at early disease stages where TDP-43 pathology has yet to be developed.

## Materials and methods

### Human samples for iPSC generation

We reprogrammed fibroblasts of two sALS patients (further referred as sALS-1-1/2 and sALS-2-1/2), two age-matched controls (Ctrl-1-1/2 and Ctrl-2-1/2, respectively) and included two additional controls (CV-B and Ctrl-3-1/2) and 2 fALS (fALS-1-1 and fALS-2-1) cases with pathogenic variants in TDP-43 in this study. The study approval for cell lines originating from Ctrl-1, Ctrl-2, sALS-1 and sALS-2 was granted by the local ethics committee (No. 4485, FAU Erlangen-Nürnberg). The 2 sALS patients did not have a family history of ALS and did not harbor known ALS-causing mutations determined by C9ORF72 repeat length analysis and exome sequencing. Both patients were diagnosed with clinically definite ALS according to the revised El Escorial criteria [40]. Patient sALS-2 presented with cortical and spinal motor neuron involvement starting in the right arm. In the latest follow-up (11 years after onset), he had been on a ventilator for the past 6 years, presenting with a revised ALS functional rating scale (ALSFRSr) of 1/48. In contrast, patient sALS-1 exhibited slower progression and an ALSFRSr of 39/48 12 years after disease onset. The use of cell lines originating from Ctrl-3, fALS-1 and fALS-2 were approved by the Institutional Review Board of the University of California, San Diego. The CV-B iPSC line, originating from Craig Venter, is publicly available and has been previously described [24]. Ctrl-3 and fALS-1 have been described previously [32]. Line fALS-2 was obtained by courtesy of Kevin Eggan and has been described before [2]. Detailed clinical information and reprogramming method for each line is stated in Online Resource Table 1. For the hereditary spastic paraplegia with pathogenic variants in SPG11 (SPG11-HSP) cases, 6 lines from 3 SPG11-HSP patients (UKERi4AA-S006, UKERi4AA-S-14A, UKERiK22-S-001, UKERiK22-S-003, UKERiG7G-S-001, UKERiG7G-S-008) and 6 lines from 3 Ctrls (UKERi33Q-S-006, UKERi33Q-R-106, UKERi82A-S-004, UKERi82A-S-022, UKERi55O-S-002, UKERi55O-S-004) were used that have been previously characterized [25, 53]. Additionally, an isogenic pair of wt HuES6 and a homozygous SPG11 knock-out was used that has been previously characterized [53]. Pluripotency as well as normal karyotype was confirmed in all iPSC lines.

### Induced pluripotent stem cell (iPSC) culture

iPSC were derived from fibroblasts and reprogrammed using Yamanaka factors [27, 62]. They were cultured on Matrigel-coated plates using mTESR1 or mTESR plus at 37 °C with 5% CO_2_. The media was exchanged every 24 hours for mTESR1 and every 48 hours for mTESR plus. When iPSCs reached about 80-90% confluency, the cells were passaged using Accutase and plated in a 1:3 to 1:6 ratio with mTESR1 + 10μM ROCK-inhibitor (RI) or with ReLeSR in a 1:3 to 1:6 ratio with mTESR+. The media was changed the day after passaging.

### Generation of NOVA1 knock out iPSC

The CV-B iPSC line was chosen to generate NOVA1 K.O. Two adjacent gRNAs targeting exon2 of NOVA1 (gRNA 1: GGATCTATAATTGGGAAGGG; gRNA 2: GGACTTAGACAGCTTGATGG) were cloned into the lentiCRISPR v2 backbone (Addgene plasmid #52961) [57]. The media was aspirated from CV-B iPSC growing in a 6-well format and 1ml of OptiMEM was given to the well. The two gRNA plasmids were jointly transfected (3ug per gRNA plasmid) in CV-B iPSC using lipofectamine 3000. After 2h, the OptiMEM solution was aspirated and mTeSR plus was added. To select for transfected cells, 24h after transfection, the media was supplemented with 0.5μg/ml puromycin, 48h after transfection with 1μg/ml puromycin and 72h after transfection with 0.25μg/ml puromycin and 96h after transfection, puromycin was withdrawn from the media. After 3d recovery, the cells were dissociated with Accutase and 2000 cells were plated onto a 6-cm dish with mTeSR plus containing CloneR. Two days after, the media was changed to mTeSR plus and cultured for an additional 10 days until iPSC clones become clearly visible. The clones were then picked into a 96-well plate that was duplicated 4 days later. One 96-well plate was lysed in QuickExtract buffer (Lucigen) for genotyping using GoTaq2x MasterMix and two primers flanking the two gRNAs (fwd: CCCAGTGCTTTAGTTGCTGT; rev: TCTTCACCATTGCACCTACCT). Candidate clones were identified by a reduction in PCR product size. All clones were expanded, checked for expression of pluripotency markers, and cryobanked. The presence of frameshift mutations was confirmed via Sanger sequencing of the PCR products. As controls, we used clones that underwent the CRISPR/Cas9 process but where no genome editing was achieved.

### Motor neuron (MN) differentiation

A dual-SMAD inhibition-based protocol [10] was used to generate MNs (Online Resource Fig. 1a) as described previously [32, 42, 44]. Briefly, iPSC were dissociated into single cells using Accutase. After cell counting, 2x10^5^ cells per cm^2^ (i.e. 2x10^6^ cells for one well of a six well plate) were plated on a Matrigel-coated well (17.5µg/cm^2^ of Matrigel in DMEM/F12) with mTESR1 or mTESR plus supplemented with 5μM RI. Around 24h later, the cells reached 95% confluency. For the differentiation into MN, a basal-media, further referred as N2B27 (DMEM/F12, 1xN2, 1x B27, 100μM ascorbic acid, 1xPen/Strep), was used for culture and dilution of compounds. From day one to day six, the cells were incubated with N2B27 supplemented with 1μM Dorsomorphin (Dorso), 10μM SB431542 (SB), 3μM CHIR99021 (CHIR). From day 3 on, 5μM RI were supplemented and the media volume was gradually increased (0.2 ml/cm^2^ on days 1 and 2, 0.3 ml/cm^2^ on days 3 and 4, and 0.4 ml/cm^2^ on the following days). The media was exchanged on daily basis. From day six to day 15 the cells were incubated with 0.5xN2B27 with Dorso, SB, RI and, in addition, 1.5μM retinoic acid (RA) and 200nM of Smoothed agonist (SAG) and 5uM RI with 0.416 ml/cm^2^. When the cells reached the stage of MN progenitors (MNPs, day 15 of differentiation), the MNPs were dissociated into single cells using Accutase. 0.5x10^6^ cells/ml were plated on poly-D lysine and laminin (PDL/Lam)-coated 6-well plates in N2B27 with RA, SAG, RI, and 2ng/ml of brain-derived neurotrophic factor (BDNF), glial-derived neurotrophic factor (GDNF), and ciliary neurotrophic factor (CNTF), respectively. This combination of growth factors will be referred to as neurotrophic factors (NFs) further on. At this point the cells were frozen in vials containing handy cell numbers for future use (1x10^6^, 5x10^6^ or max. 10x10^6^ cells). The appropriate amount was centrifuged again and resuspended in 500μl N2B27 and 500μl of a freezing solution consisting of 80% FBS and 20% DMSO. The vials were put in a freezing container at −80 °C for three hours. Afterward, the vials were immediately transferred into liquid nitrogen or −150°C . 

For further culture, the media containing RA, SAG, 2μM RI and NFs was exchanged completely every other day until day 22. On day 22 of MN differentiation, the media was switched to N2B27 with NFs, 2μM RI and the γ-secretase inhibitor DAPT (2μM). On day 24, the cells were fed with the same media again. From day 25 on the cells were cultured until day 30 solely with N2B27 supplemented with NFs and 2μM RI.

To ensure an appropriate cell densities for immunofluorescence (IF) staining and imaging, a portion of the cells were passaged with Accutase between day 22 and 25 for each differentiation round on the same day. The procedure follows the dissociation at day 15 as explained above.

### Lentivirus production and infection of iPSC-derived MNs

Lentivirus was produced as described previously [32]. Plasmids compatible for lentiviral packaging were generated by assembling NOVA1 ORF or EGFP ORF with a P2A-PuroR DNA fragment into a CAG promoter-containing backbone [71]. Briefly, 2nd generation lentivirus packaging plasmids were used to produce VSV-G pseudotyped lentivirus containing CAG-ORF-V5-P2A-PuroR in Lenti-X-293T cells with PEI as a transfection (10µg VSV-G, 15µg psPAX2, 20ug ORF plasmid) reagent in 15-cm dish formats. Five hours after transfection, the media was changed to DMEM/F12+Glutamax containing 10% FBS. 24h and 48h after transfection the media was harvested and pooled together. The media was then centrifuged at 1,000g for 5 mins to remove cell debris. Lenti-X-concentrator was used according to the manufacturer’s instructions to concentrate the virus by 100x. The virus stock was stored at −80 degrees. Different viruses used for a given experiment were always prepared together.

For infection of NOVA1 and EGFP overexpression in iPSC-MN resulting in the generation of the analyzed RNA-seq datasets, 30µl of virus concentrate was applied per well of a 24-well dish with MNs at day 22 of differentiation. At day 24 of differentiation 1μg/ml puromycin was added to the media to get rid of non-transduced cells and kept in the media till day 29 of differentiation. At day 26, a second round of lentivirus infection with 30μl of virus concentrate was applied to get a sufficient degree of overexpression. At day 30, one well of each condition was lysed in TriZOL for RNA and another one in RIPA buffer to confirm overexpression by Western blot.

### FACS analyses

All FACS experiments were performed with 6 control and 6 ALS lines in parallel. For flow cytometry the MNPs or MNs were dissociated with Accutase for 30 min at 37 °C and resuspended in FC buffer (2% FCS, 0.01% sodium azide in PBS). Cells were dispensed into 5 ml tubes (Sarstedt) at 500,000 cells per well. For intracellular antigens, cells were fixed and permeabilized with 100µl BD Fixation/Permeabilization Solution (BD Bioscience) for 10 minutes, then 1ml of BD Perm/Wash Buffer is added and the cells are incubated for 5 minutes and subsequently centrifuged at 1500 rpm for 3min. For intracellular staining of MNPs, an OLIG2 antibody (AB9610, Millipore, 1:100) was incubated in BD Perm/Wash Buffer for 30 minutes and after washing, the cells were incubated with an AlexaFluor-488 anti-rabbit IgG (1:500), anti PAX6-APC and anti NESTIN-PerCp-Cy5.5 for an additional 30 minutes. After a wash step, the cells were resuspended in 350µl FACS buffer containing DAPI (1μg/ml). For intracellular staining of neurons, the cells were stained with anti bIII-Tubulin (NB600-1018AF405, NovusBio, 1:100) or anti-ISL1 (562547, BD Bioscience, 1:200) for 30min. The gating strategy for all FACS experiments included undifferentiated iPSC as a negative control to validate specificity of the antibody since iPSC did not express any of the genes of interest at relevant levels on RNA level as determined by an in-house RNA-seq dataset from iPSC (RPKM OLIG2: 0; RPKM PAX6: 0.06; RPKM ISL1: 0.01; RPKM bIII-tubulin: 0.002) [29]. Additional controls included applying an antibody solution missing one antibody in the full cocktail (“minus 1 control”) that were used to determine potential bleed-through of the fluorophores. The flow cytometry experiments were performed with a Cytoflex S (laser 405nm, 488nm, 561nm, 638nm; Beckman Coulter) and analyzed with the CytExpert 2.4 software.

To determine cell death via FACS, we used a commercially available kit that uses a fluorescent 660-DEVD-FMK caspase-3/7 inhibitor reagent (ab270785, abcam) and a fixable cell permeability dye (Live-or-Dye, 32008-T, Biotium). The caspase assay and Live-or-Dye assay reagents were dissolved in 50µl DMSO, respectively and aliquoted and stored at -20 degrees. For the assay, MN were grown in 24-well plates. At the day of analysis, the media was aspirated from the plate and 150µl DMEM/F12+Glutamax containing 0.48µl 660-DEVD-FMK caspase-3/7 inhibitor reagent and 0.15μl Live-or-Dye assay were applied. The cells were then incubated for 45 minutes at 37 degrees C. Subsequently, the cells were dissociated, fixed and stained as stated above. To correctly determine bleed through, single incubation controls (either with 660-DEVD-FMK caspase-3/7 inhibitor reagent or Live-or-Dye assay) were used. The number of Casp3/7^+^ISl1^+^Live-or-Dye^-^ cells vs. Casp3/7^-^ISl1^+^Live-or-Dye^-^ were determined as the final readout.

### Immunofluorescence staining of cultured cells

Cells were fixed in 4% paraformaldehyde (PFA) for 10 minutes at room temperature and subsequently washed 3x with PBS for 3 mins each. A total of 6 different ALS and 6 different Ctrl lines were processed for all stainings in parallel.

The cells were permeabilized and blocked in 0.3% Triton X-100 and 5% donkey serum in PBS for 30 mins at room temperature. Afterward, the cells were incubated with primary antibodies (ISL1: 39.4D5-s, DSHB, 1:250; beta-III-Tubulin: ab18207, Abcam, 1:1000; NOVA1, ab183024, Abcam, 1:500; TDP-43, A303-223A, Bethyl, 1:1000) at 4 °C overnight. After washing, incubation with secondary antibodies and nuclei staining using 1µg/ml DAPI was performed. The slides were mounted using Mowiol solution.

Imaging was performed with a Zeiss Observer.Z1 including Apotome technology. For the quantification of ISL1 positive cells, a custom ImageJ script, counting at least 250 neurons per sample was used. For determining NOVA1 and TDP-43 intensities in iPSC-MN, 15 positions with relatively sparsely located ISL1-positive cells were imaged using a 63x objective and 9 Z-stacks with 0.5μm thickness. Using Zen blue software (Zeiss), raw apotome phase correction was performed and the Z-stacks were merged using extended depth of focus (maximum intensity projection) using default conditions and exported as .tiff files. CellProfiler [45] was used for subsequent image analysis. Briefly, all nuclei and ISL1 cells primary objects were identified by DAPI and ISL1 images, respectively (Otsu thresholding method with 3 classes). Neuron somas were identified using the propagation method on the log-transformed beta-III-tubulin images (Otsu thresholding method with 3 classes, middle class assigned to the foreground). To reduce falsely positively assigned objects, somas with a size > 30,000 pixels were not further considered. Objects were related with each other to only consider further ISL1 positive nuclei and the corresponding somata. By subtracting the nuclear area from the soma area, the cytoplasmic area was determined. Using the measure intensity plugin, cytoplasmic and nuclear intensity of our proteins or interest in MNs was measured. A median filter (window: 5) was applied before intensity measurement to reduce salt and pepper noise. For NOVA1, the first 28 MNs per line were considered and for TDP-43 20 MNs per line were considered.

### Protein fractionation

The MNs, which form a dense network of cells, were washed with warm DPBS. The sheet of cells was transferred to a 1.5ml Eppendorf tube and centrifuged. After removing the supernatant, the pellet weight was determined. For 1μg cell pellet, 4μl of ice-cold RIPA buffer were added to facilitate the lysis of the cells. The cell lysate was sonicated for five minutes with 30s on/off on a low intensity level using a Bioruptor. Afterward, the lysate was centrifuged at 100,000g for 30min at 4 °C. This supernatant represents the soluble protein fraction. The pellet was washed with ice-cold RIPA once and then resuspended in 4μl urea buffer (7M urea, 3M thiourea, 4% CHAPS, 30mM Tris) per original μg of pellet weight. Subsequently, the suspension was sonicated using a Bioruptor under the same conditions as described before. The sonicated suspension is centrifuged at 100,000g for 30min at 4 °C again. The resulting supernatant reflects the insoluble fraction. To each fraction, loading buffer and DTT (final concentration of 100mM) was added and the samples were incubated at 55 °C for 30min. The samples were then used for Western blotting. Western blot band intensities were quantified by densitometry and normalized to total protein determined from separately run Coomassie-stained gels.

### Western blot

For regular total cell lysates, cells were lysed in Radio-immunoprecipitation buffer (RIPA) followed by sonication with a Bioruptor and the protein concentration was estimated by bicinchoninic acid (BCA) assay. Equal concentrations were applied. All immunoblots were run on 4-12% Bis-Tris gels with NuPAGE MOPS running buffer for 90 minutes at 180V. Proteins were transferred to a PVDF membrane with 10% methanol in NuPAGE transfer buffer at 30V overnight at 4 °C. The membrane was then blocked for 1h in 5% bovine serum albumin (BSA) in TBS-T and primary antibody (NOVA1: ab183024, Abcam, 1:2000; NOVA2: 55002-1-AP, ProteinTech, 1:1000; RBFOX2: A300-864A, Bethyl Laboratories Inc., 1:1000; RBFOX3: ab177487, abcam, 1:1000; ELAVL4: sc-48421, Santa Cruz Biotech., 1:1000; FXR2: MA1-16767, ThermoFisher, 1:1000; GAPDH: CB1001, Millipore, 1:5000; TDP-43: A303-223A, Bethyl Laboratories Inc., 1:5000) was incubated over night at 4°C. Afterward, the membrane was washed twice for approximately seven minutes in 5% BSA in TBS-T and then incubated for one hour with the secondary HRP-conjugated antibody for one hour at room temperature. The membrane was washed three times with TBS-T and incubated in the dark with ECL solution. Film development was performed in the dark with various exposure times. For quantification of Western blots, densitometric analysis was performed using Fiji.

### Mass spectrometry

Label-free quantification of peptides from urea-soluble fractions (representing insoluble protein; no DTT and loading buffer added) was done using a Thermo Scientific Orbitrap Fusion instrument at the Core Facility for Medical Bioanalytics at the University Hospital Tübingen. Peptide identification and assignment was performed using MaxQuant [13]. LFQ intensities representing peptide quantities were used for further calculations of proteins enriched in the urea fraction. Controls (CV-B, Ctrl-3-1, Ctrl-1-1, Ctrl-1-2, Ctrl-2-1, Ctrl-2-2) were compared to ALS samples (sALS-1-1, sALS-1-2, sALS-2-1, sALS-2-2, fALS-1-1, fALS-2-1). For further calculations, +1 was added to all values to avoid divisions by 0. A fold changed was computed (mean(LFQ intensity+1)_ALS_ / mean(LFQ intensity+1)_Ctrl_). To test for statistical significance, Welch’s test was performed on log_2_-transformed LFQ intensity+1 values. A *P* value below 0.05 was considered as significant. Additionally, we used a fold change cutoff of greater than 1.5. The SciPy package was used for statistical calculations and seaborn package for visualization in Python 3.0.

### RNA extraction

TriZOL was used for RNA extraction according to the manufacturer’s instructions. All steps were performed on ice or 4 °C. The RNA was resuspended in DNase/RNase free H_2_O. For obtaining ultrapure RNA without any residual phenol-chloroform contamination, the Zymo RNA Clean & Concentrator-5 kit was used.

### RNA-seq library preparation

For the RNA-seq dataset sALS vs. Ctrl of this study, the RNA-seq libraries were prepared using the Illumina TruSeq Stranded mRNA Library Prep Kit and performed according to the manufacturers protocol. In total, 1μg of RNA was used as input and poly-A-selection was performed. The library was quantified using a Tapestation (Agilent Technologies 2100 Bioanalyzer). To ensure library quality was suitable the fragment size was checked. The prepared library was sequenced on an Illumina HiSeq4000 instrument at the IGM Genomics Center at UCSD. Libraries were sequenced in 100bp, paired-end mode. For all other RNA-seq datasets generated in this study (fALS vs. Ctrl, NOVA1 overexpression vs. EGFP overexpression, NOVA1 K.O. vs. NOVA1 wt, SPG11-HSP vs. Ctrl), library preparation and sequencing was performed at Genewiz Germany GmbH with Illumina Stranded mRNA library preparation and sequencing in 150bp paired-end mode. All libraries were sequenced to a depth of > 40x10^6^ reads.

### Public datasets

Additional RNA-seq data from ALS patients were obtained from the AnswerALS consortium (http://data.answerals.org). Data present by December 2020 were downloaded. To ensure adequate comparability of patients and controls, stringent inclusion criteria were set (Online Resource Fig. 3b). All ALS patients considered were diagnosed with at least clinically probable, laboratory supported ALS and presented lower motor neuron signs in at least 3 different sites. Furthermore, ALS patients were subdivided according to their C9ORF72 hexanucleotide repeat expansion (C9) status and sex. Additionally, for C9-negative ALS patients and Controls, a family history of neurological disorders was excluded. Due to an insufficient number of male individuals that passed these criteria, this resulted in datasets from 9 sALS, 5 C9-ALS and 9 Ctrl individuals (consisting of female individuals only) that were used for further analysis.

For datasets used for investigating TDP-43 related changes in NOVA1 expression, 3 TDP-43 depletion datasets (GSE121569 [31], GSE122069 [46], GSE27394 [52]) and 2 datasets with cytoplasmic TDP-43 (GSE65973 [3], GSE157467 [41]) were used.

### RNA-seq mapping and alternative splicing analysis

All RNA-seq datasets were analyzed in the same fashion unless stated otherwise. The RNA-seq reads were adaptor trimmed using Cutadapt (version 1.10) [43], aligned to hg19 (GRCh37) with STAR (version 2.5.0b) [18] and sorted using samtools (version 0.1.18) [34]. The aligned reads were assigned to the gencode annotation (version 19) using featureCounts [35] and Reads Per Kilobase of transcript, per Million mapped reads (RPKM) were calculated.

For differential splicing rMATS (version 4.1.0) was used [60] with the following specifying flags: -b1 *samples-in-control-condition.bam* -b2 *samples-in-target-condition.bam* -t paired --variable-read-length --anchorLength 1 --tstat 6 --novelSS --libType fr-firststrand. The output file considering the junction counts only was used for further analysis. Due to order of the assignment of groups in -b1 and -b2, negative value of the InclusionLevelDifference reflects an inclusion of a given exon in the samples of the target condition and a positive value of the InclusionLevelDifference reflects an exclusion of a given exon in the samples of the target condition.

All downstream analysis were performed in Python 2.7. Only exon junctions that were covered with at least 10 counts in each sample of a given dataset were considered. A unique index was generated, referring to a specific AS event with the aim to identify the exact same exon junction in separate rMATS analyses. For pair-wise comparisons of datasets, the files were joined and only exons that passed the coverage threshold in both datasets were considered. An exon was called as differentially alternatively spliced in each dataset if the FDR was below 0.05 and the absolute value of the InclusionLevelDifference was more than 0.1. The overlap of differentially spliced events was visualized with the Venn function in matplotlib library. The significance of the overlap and odds ratio (OR) was determined by Fisher’s exact test using the stats module in SciPy. The value and significance of the Spearman correlation analysis of the InclusionLevelDifference in two given datasets was also computed with the stats module in SciPy. For k-means clustering, the k-means method from the sklearn.cluster module from sciki-lean [51] was used (specifications: init=’ranodm’, n_clusters=8, n_init=10, max_iter=300, random_state=42). AS events significant in any of the four ALS datasets were clustered into 8 clusters according to the inclusion value differences in the four ALS datasets, the SPG11-HSP, and the NOVA1 o.e. and K.O. dataset.

### eCLIP-seq library preparation and raw data processing

The standard eCLIP-procedure was performed for TDP-43 [67, 68] and the single-end sequencing version of the assay was applied to the eCLIP-seq experiments for NOVA1, NOVA2 and RBFOX2 [66] with some modifications to adapt it to *in vitro* cultured MNs. Briefly, for iPSC-derived MNs, one full 10-cm culture plate (containing ~5x10^7^ cells) was UV cross-linked (254 nm, 400 mJ/cm^2^), flash frozen and stored at -80°C. For eCLIP-seq, the cells were lysed, sonicated and cleared by centrifugation (15min at 15,000g, 4 °C). This lysate was used for further downstream preparations. The antibody (TDP-43: A303-223A, Bethyl Laboratories, Inc.; NOVA1: ab183034, abcam; NOVA2: 55002-1, ThermoFisher; RBFOX2: A300-864A, Bethyl Laboratories, Inc.) was bound on Dynabeads and incubated for 2h at room temperature with the pre-cleared lysate. Afterward, the beads were washed and the barcoded 3’RNA linkers were ligated on the bound RNA on the beads. The IP and inputs were loaded on a gel and transferred onto nitrocellulose. A 70 kDa fragment was excised from the nitrocellulose membrane for each lane starting from the size of the protein of interest to capture most of the IPed RNA and a size-matched input (input) as a control. Membrane fragments were treated with protease and released RNA was extracted using Zymo RNA Clean & Concentrator-5 columns. The RNA was reverse transcribed with a primer specific to the ligated RNA adapter. Excess primers were digested with ExoSAP, the RNA was removed by NaOH treatment, the cDNA was purified using MyONE Silane beads, and 5’ adapters were ligated on the cDNA. After quantification, the library was amplified using Q5 PCR mix (NEB) and size selected. The exact libraries were quantified using a D1000 tape on an Agilent Bioanalyzer. For the TDP-43 eCLIP-seq experiments, the libraries were sequenced in 50bp paired-end mode, while NOVA1, NOVA2 and RBFOX2 eCLIP-seq experiments were 75bp single-end.

To obtain a peak file with enrichments over inputs with log_2_(FC) and *P* values, a standard peak calling pipeline was use as described previously [68] (code available on github: https://github.com/YeoLab/eclip). Briefly, the adapters were trimmed and the reads were aligned to the UCSC hg19 genome built using STAR [18]. A custom script performed removal of PCR duplicates and CLIPper [39] was used to call peaks. To calculate enrichments over input, reads in IP and SMI were compared at regions identified by CLIPper in the IP sample. Fold change and *P* values were calculated by Fisher’s exact test. A threshold was applied to call significantly enriched peaks. For TDP-43, the threshold was set to log_2_(FC) > 5 and a –log_10_(*P* value) > 5 and for NOVA1, NOVA2 and RBFOX2 the threshold was set to log_2_(FC) > 3 and a –log_10_(*P* value) > 3.

### Region analysis and enrichment of k-mers

To determine the distribution of transcript regions within the significantly enriched peaks of an eCLIP-seq dataset, the annotator tool was used (https://github.com/byee4/annotator). To analyze sequence preferences in the significantly enriched peaks, we performed k-mer analysis (https://github.com/byee4/clip_analysis). The occurrence of each possible 4-mer or 6-mer was calculated for a peak file and a *Z* test was performed to calculate the frequency of each 4-mer or 6-mer over the background.

### Determination of enrichment of eCLIP-seq peaks in AS events

To determine if an eCLIP-seq peak was present in an exon junction, the rMATS output of interest was converted into a bed format and intersected with the significant eCLIP-seq peak file using pybedtools (-u True) [14]. The different regions within the exon junction were also extracted from rMATS, acknowledging that upstream regions of exon junctions on the negative strand will be displayed as downstream regions by rMATS. The statistical significance of the enrichment was computed using hypergeometric test with all events that passed the coverage threshold as the background.

### Human postmortem tissue immunofluorescence staining and imaging analysis

All *postmortem* CNS tissues were acquired by way of an Investigational Review Board and Health Insurance Portability and Accountability Act compliant process. All samples (*n *= 9 sALS; *n *= 7 Ctrl, including 2 AD patients) had *postmortem* intervals <8 h. The sALS tissues were from patients who had been followed during the clinical course of their illness and met El Escorial criteria for definite ALS. Genetic variants that are known to cause ALS were excluded.

Six micrometer thick tissue sections from lumbar spinal cord were cut from a block of formalin-fixed paraffin embedded tissue and deparaffinized using CitriSolv (2x15mins) and a series of gradually decreasing ethanol wash steps (2x100%, 1x90%, 1x70% and 1x0%, 5 minutes each). Subsequently, antigen retrieval was performed using high pH antigen retrieval solution at 120 °C for 15 minutes. The staining vessel containing the samples was allowed to cool down on the benchtop for 30 minutes, washed 2x for 5 minutes with PBS and permeabilized in 0.2% Triton-X-100 in PBS for 10 minutes. Afterward, the tissue was blocked with 2% FBS in PBS for 60 minutes followed by the primary antibody incubation in 2% FBS in PBS over night at 4 °C (TDP-43: H00023435-M01, Novus Biologica, 1:500 dilution; NOVA1: ab183034, abcam, 1:500 dilution; beta-III-Tubulin: ab107216, abcam, 1:1000). The next day, the tissue was set to room temperature for 3 minutes, then washed 3x5 minutes in PBS and blocked in 2% donkey serum with 0.2% Triton-X-100 in PBS for 30 minutes. Subsequently, the secondary antibodies were incubated for 1h at room temperature (AlexaFluor-488, anti-rabbit; AlexaFluor-555, anti-mouse; AlexaFluor-633, anti-chicken; all at 1:500 concentration). After three 5-min wash steps in PBS, the nuclei were stained with 1µg/ml DAPI for 10 min and then washed again twice for 5 min in H_2_O. To reduce autofluorescence, the samples were incubated in 0.1% (w/v) Sudan Black in 70% ethanol for 15 seconds and washed twice for 5 min each in PBS immediately after. The slides were submersed in H_2_O and mounted with ProLong Gold Antifade with DAPI (ThermoFisher).

All slides were imaged using the identical imaging conditions with a Zeiss Spinning Disc Axio Observer Z1. To promote unbiased image selection, a 10x overview of the beta-III-tubulin signal of anterior horn of the spinal cord was taken and using the tiles setup, regions containing MNs were selected and subsequently imaged with a Plan-Apochromat 63x oil magnification objective and 23x0.5um z-stacks. Using Zen software, the tiles were conjoined, and a maximum intensity projection was computed and converted to a TIFF which was used for further analysis. Imaging was performed blinded manually on a single-MN level in Fiji [58]. Only MNs with a visible nucleus as determined by DAPI were analyzed. The mean fluorescent intensity (MFI) of NOVA1 and beta-III-tubulin was measured in the nucleus, soma and lipofuscin areas in each neuron. An area was considered as lipofuscin when the typical autofluorescence pattern was observed in all channels. Additionally, the area of the three locations of interest was measured and the lipofuscin-free cytoplasmic area was calculated by subtracting the nuclear and lipofuscin areas from the soma area. The MFI of the cytoplasm was then calculated as followed: MFI_cytoplasm_ = ((MFI_soma_xarea_soma_) − (MFI_nucleus_xarea_nucleus_) − (MFI_lipofuscin_xarea_lipofuscin_))/area_cytoplasm_. Qualitatively, each MN was assessed for TDP-43 pathology, specifically if there is a loss in nuclear TDP-43 and whether there is cytoplasmic TDP-43 aggregation present and if the aggregation is dot-like or skein-like/Lewy-body-like.

Analysis of the data was performed in Python 3. Only MNs with an area_soma_ > 1000µm^2^ were considered. For the analysis between MNs within a sALS patients, the raw MFI was used and MFIs in MNs with nuclear TDP-43 and loss of nuclear TDP-43 within a single patient were considered as matched values. To compare MNs from different individuals with each other, the MFI of NOVA1 and TDP-43 were normalized to the beta-III-tubulin MFI of the lipofuscin-free soma area, with the aim to reduce inter-individual variability due to technical differences such as *postmortem* interval, variability within fixation procedure and storage time of the tissue.

### NOVA1-STMN2 expression correlation analysis in postmortem ALS tissues

To investigate changes in NOVA1 expression in ALS patient tissue with varying TDP-43 dysfunction, we used 4 publicly available sALS gene expression datasets: GSE76220 [32] and GSE18920 [55] containing spinal motor neuron RNA-seq and microarray datasets, respectively, from laser capture microdissected tissue; and GSE122649 and GSE124439, containing RNA-seq data from motor and non-motor frontal cortex [63]. For GSE76220, reads were aligned to the human genome, assigned to genes and converted to RPKM values as described before. For GSE56504, microarray gene expression values of NOVA1 and STMN2 were extracted, by only considering the probe with the highest signal for NOVA1 (3558755) and STMN2 (3104516) using GEO2R. For GSE122649 and GSE124439, the counts table provided via the GEO accession number was used and counts were transformed into reads per million (RPM) values. To correlate NOVA1 and STMN2 expression values in the different datasets, Spearman R correlation analysis was performed in Python 3 using the SciPy package and scatter blots were visualized using the lmplot function within the seaborn package in Python 3. To pool the samples of different datasets within the two anatomical regions (spinal motor neurons and frontal cortex), NOVA1 and STMN2 expression values were converted into z-scores based on the mean and standard deviation of controls in the respective dataset. Since GSE76220 and GSE18920 have a partially overlapping set of patients, the duplicated patients were omitted from the GSE18920 dataset. ALS samples were then assigned to either a STMN2 regular or a STMN2 low group by setting the z-score cutoff value at -2. Using the SciPy package in Python, statistical significance in expression in the 3 groups (Ctrl, ALS STMN2 regular, ALS STMN2 low) was computed using Kruskal-Wallis test and individual differences between two groups were determined using Wilcoxon rank-sum tests.

### Statistical analyses

GraphPad Prism 9.0 was used for generation of simple blots and computation of *P* values of simple pair-wise or grouped analyses. Wherever possible, non-parametric tests were used. For all pair-wise analyses, 2-sided Mann-Whitney *U* test was performed. To compare significance between groups consisting of multiple individuals (*postmortem* imaging), nested one-way ANOVA was used. Kruskal-Wallis test was used to determine a difference in more than 2 groups and Dunn’s post hoc test was applied to identify differences between individual groups. A mixed effects model analysis was performed when a significance was computed to determine the difference between more than one categorical independent variable when matched values were compared. 2-way ANOVA was used for similar comparisons of unmatched samples. *P* value < 0.05 was considered significant (*P value < 0.05; **P value < 0.01; ***P value < 0.001, ****P value < 0.0001).

## Results

### Mass spectrometry identifies insoluble candidate RBPs in ALS

**Fig. 1:**
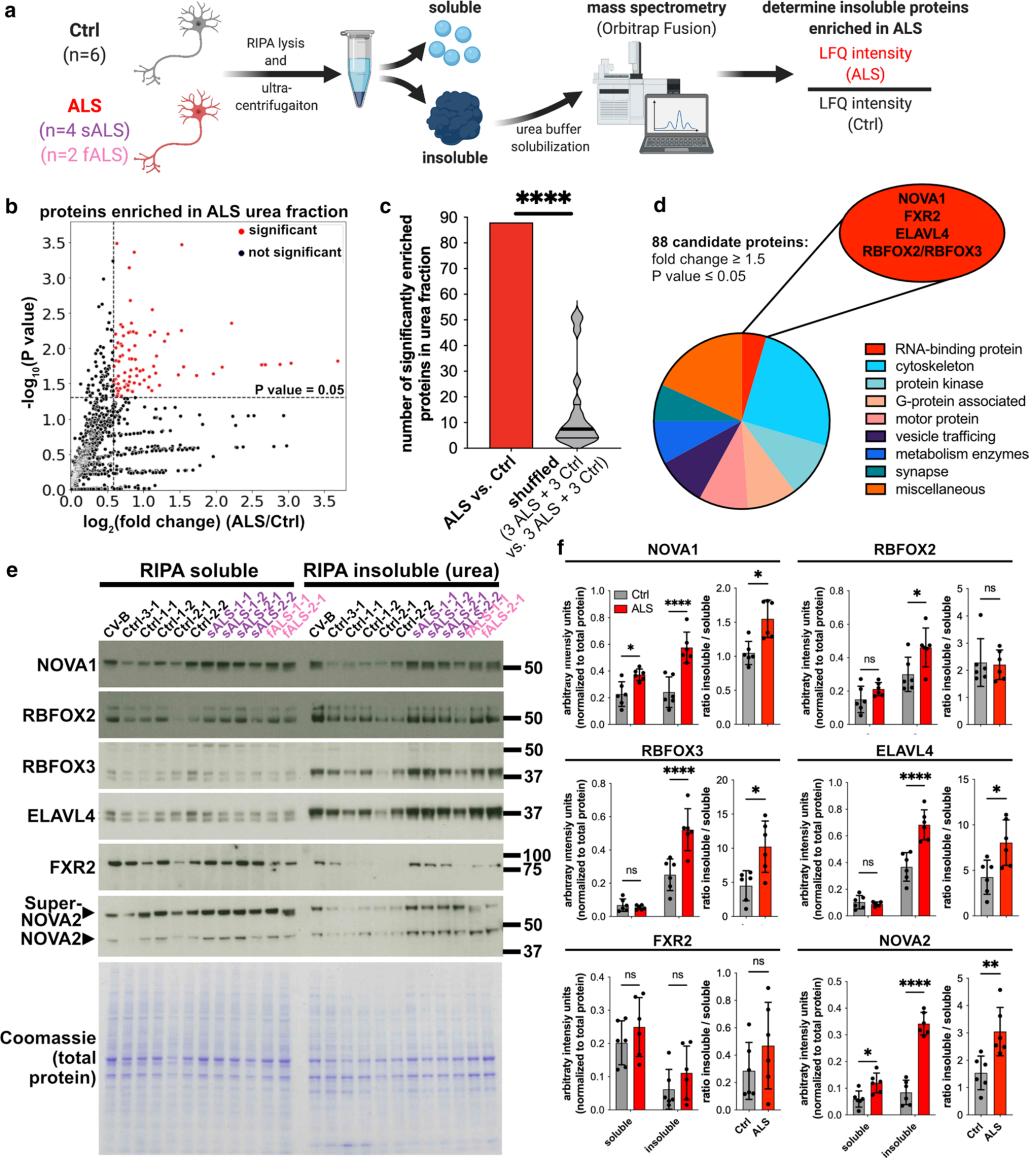
Protein fractionation of iPSC-derived motor neurons followed by mass spectrometry analysis identifies RBPs with increased insolubility in ALS. **a** Schematic illustrating the experimental and analysis workflow. **b** Scatter plot presenting log_2_(fold change) ALS/Ctrl (x axis) and significance (-log_10_(*P* value)) (y axis). Significant events are shown in red (horizontal dashed line: *P* value = 0.05; vertical dashed line: fold change = 1.5; *n *= 6 ALS, *n *= 6 Ctrl) **c** Bar graph showing number of proteins significantly enriched in ALS samples vs. Ctrl (red bar), or when iteratively shuffled (3 ALS samples and 3 Ctrls were compared to the respective remaining 3 ALS samples and 3 Ctrls, gray violin plot). Significance was determined by Mann-Whitney *U* test against a theoretical median of 88. **d** Pie chart highlighting protein classes of the 88 candidate peptides enriched in ALS insoluble fractions. RNA-binding proteins are highlighted in the red ellipse. **e** Western blots of candidate RBPs in soluble and insoluble fractions and corresponding Coomassie-stained gel (*n *= 6 ALS, *n *= 6 Ctrl). The SuperNOVA2 isoform is produced from an upstream alternative start codon [56]. **f** Densitometric quantification of Western blots normalized to the Coomassie controls (left graph for each protein) and ratio insoluble/soluble protein (right graph for each protein) for NOVA1, RBFOX2, RBFOX3, ELAVL4, FXR2, and NOVA2. Statistical significance was determined by 2-way ANOVA (left graph) and Mann-Whitney U test (right graph). *P* values for 2-way ANOVA were corrected using FDR. Data presented as mean ±SD (gray bars: Ctrl; red bars: ALS) and individual values (dots) (*n *= 6 ALS, *n *= 6 Ctrl). *P value < 0.05; **P value < 0.01; ***P value < 0.001; ****P value < 0.0001

To discover RBPs with increased insolubility in a human ALS model, we applied a well-established dual-SMAD inhibition-based protocol [21, 41, 42, 44] to generate iPSC-MN from six control (Ctrl) iPSC lines, from four iPSC lines originating from two sALS patients, and from two iPSC lines originating from fALS patients with pathogenic variants in the *TARDBP* gene (Online Resource Table 1; Online Resource Fig. 1a). No difference in differentiation capacity was observed (Online Resource Fig. 1b-g), resulting in ~40% ISL1^+^ MN (Online Resource Fig. 1g), comparable to numbers observed in large scale MN differentiation studies [6]. The susceptibility of ALS MN to sodium arsenite-induced stress was not changed (Online Resource Fig. 1h, i). TDP-43 insolubility is a hallmark of ALS [48]. Next, we aimed to investigate TDP-43 insolubility in this model. We fractioned iPSC-MN by lysis in radio-immunoprecipitation assay (RIPA) buffer, followed by ultracentrifugation and solubilization of RIPA insoluble proteins in urea buffer. The ultracentrifugation-cleared RIPA insoluble protein fraction is widely used to study protein insolubility in the context of neurodegeneration [50, 69]. TDP-43’s insolubility levels were unchanged in ALS iPSC-MN (Online Resource Fig. 2a-c). TDP-43 localization deficits were also not observed (Online Resource Fig. 2d-f), in line with recent observations in ALS iPSC-MN [31]. Next, we asked if there are other RBPs that exhibit an increased insolubility in our ALS iPSC-MN. Label-free mass spectrometry of the insoluble protein fraction was utilized to identify proteins that are insoluble in sALS and fALS, relative to control iPSC-MNs (Fig. 1a). Gene ontology (GO) analysis of the 100 proteins (top 2.9% of all detected proteins) with the highest label free quantification (LFQ) intensities in controls (Online Resource Fig. 2g) revealed that ‘unfolded protein binding’ (corrected *P* value = 7.95x10^−16^) and ‘structural constituent of cytoskeleton’ (corrected *P* value = 1.47x10^-10^) were among the 10 most significantly enriched GO terms, indicating enrichment of insoluble proteins (Online Resource Fig. 2h). Principle component analysis of the insoluble fractions did not distinguish ALS from control samples, suggesting that the overall insoluble proteome is not changed (Online Resource Fig. 2i). At a threshold of *P* value ≤ 0.05 (Welch’s *t* test) and fold change ≥ 1.5, we identified 88 proteins enriched in the insoluble fraction in ALS samples relative to Ctrl (Fig. 1b). When the sample labels were randomly shuffled, we observed an average of 7.5 proteins (~12-fold lower) as differentially enriched at the same statistical thresholds, indicative of an ALS-specific protein insolubility pattern (Fig. 1c). The 88 candidate proteins included cytoskeletal components and motor proteins, functional categories associated with prominent ALS *in vitro* phenotypes [1, 20, 22, 26, 33] (Fig. 1d). This included prominent examples, such as KIF5A that has been genetically linked to ALS [8, 49], and where pathogenic variants in the gene have recently been described to lead to increased KIF5A protein insolubility [5], emphasizing the validity of our approach.

Notably, also 5 RBPs, NOVA1, ELAVL4, FXR2, RBFOX2, and RBFOX3 were enriched (Fig. 1d). The NOVA1 paralog NOVA2 was significantly enriched (*P* value = 0.03) but did not meet our enrichment threshold (fold change = 1.36). Interestingly, insoluble TDP-43 protein was not significantly different in ALS and Ctrl (*P* value = 0.98; fold change = 0.97), as expected from our previous analysis. Since our primary point of interest are RBPs, we performed Western blot analysis of the candidate RBPs. We confirmed the increase in insolubility of NOVA1, NOVA2, ELAVL4, RBFOX2 and RBFOX3 (Fig. 1e and f). The soluble protein levels of NOVA1 and NOVA2 were also increased (Fig. 1f). Interestingly, also the ratio of insoluble to soluble protein was increased for NOVA1, NOVA2, RBFOX3 and ELAVL4 (Fig. 1f). In conclusion, we identified five RBPs with elevated insoluble protein levels in ALS-iPSC-MNs.

### NOVA1, NOVA2 and RBFOX2 are associated with alternative splicing in ALS

**Fig. 2:**
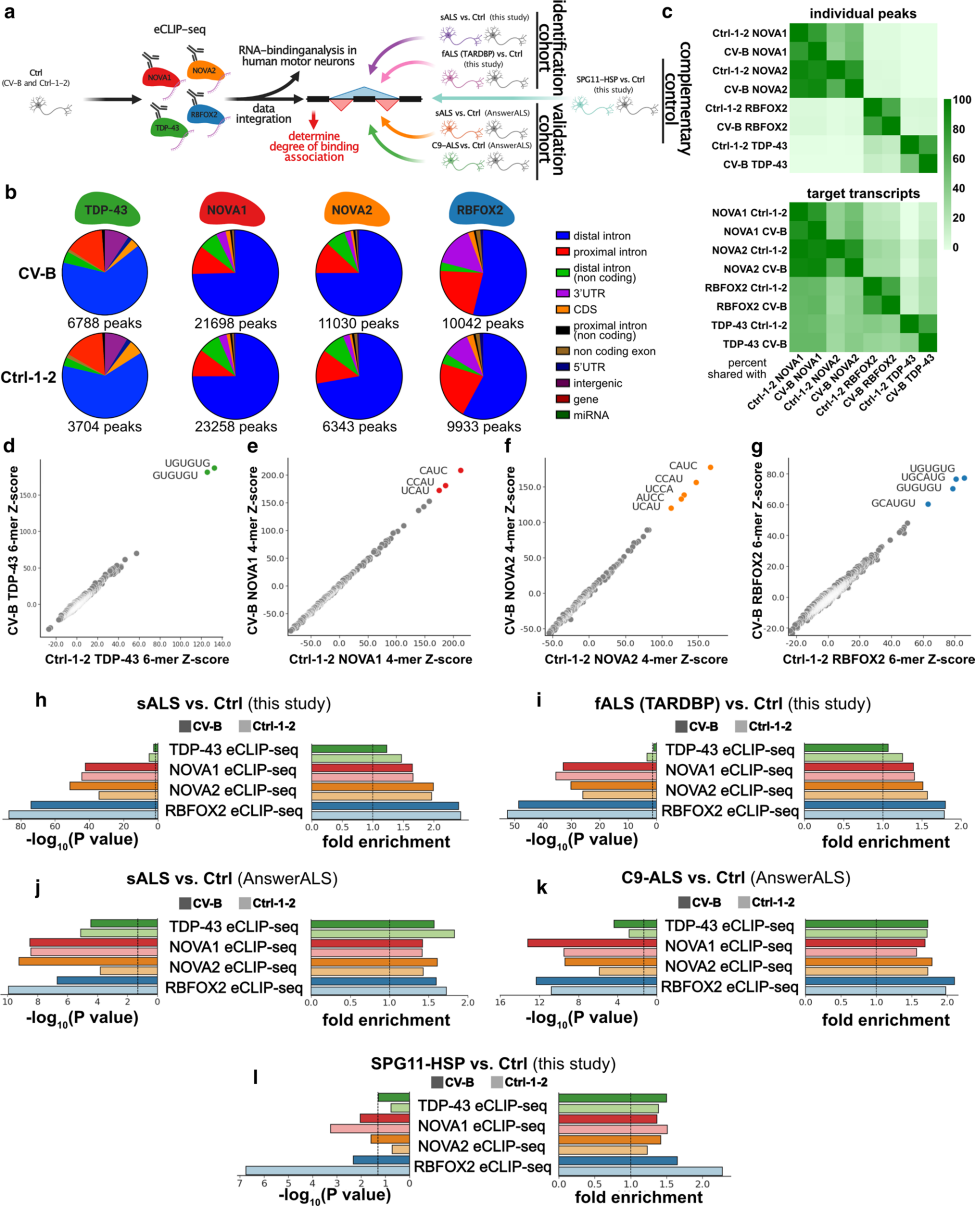
Binding of NOVA1, NOVA2 and RBFOX2 is associated with AS events that are differentially regulated in ALS. **a** Schematic illustrating the experimental and analysis workflow *n *= 2 Ctrl per eCLIP). **b** Pie charts depicting distribution of gene regions bound by eCLIP-seq peaks in a dataset. The total number of significant peaks is shown below the individual pie charts. **c** Heatmaps illustrating the percentage of individual peaks (top) and target transcripts (bottom) shared between the indicated eCLIP-seq datasets. **d**–**g** Scatter plot presenting k-mer enrichments as Z-scores of **d** TDP-43 (6-mers), **e** NOVA1 (4-mers), **f** NOVA2 (4-mers), and **g** RBFOX2 (6-mers) in the Ctrl-1-2 (x axis) and the CV-B (y axis) dataset, respectively. k-mers with highest enrichment are colored. **h**–**l** Bar graphs illustrating enrichment significance (left graph, −log_10_(*P* value))) and fold-enrichment (right graph) of TDP-43 (green), NOVA1 (red), NOVA2 (orange) and RBFOX2 (blue) eCLIP-seq experiments in CV-B (dark shading) and Ctrl-1-2 (light shading) at AS events called in the **h** sALS vs. Ctrl dataset generated in this study, **i** fALS vs. Ctrl dataset generated in this study, **j** sALS vs. Ctrl dataset from AnswerALS, **k** C9-ALS vs. Ctrl dataset from AnswerALS, and **l** SPG11-HSP vs Ctrl generated in this study. Dashed vertical lines indicate significance thresholds (*P* value = 0.05 and fold enrichment=1). Hypergeometric test was used for calculation of significance. All detected events that passed the coverage threshold in the RNA-seq datasets were used as background

NOVA1, NOVA2, RBFOX2 and TDP-43 are well-known regulators of AS. To identify their RNA-binding sites, we performed enhanced cross-linking immunoprecipitation (eCLIP) [67] for each RBP in two Ctrl iPSC-MN lines (CV-B and Ctrl-1-2) independently and sequenced the libraries of IPs and size-matched inputs at a depth of > 7 million reads each (Fig. 2a). Expectedly, NOVA proteins bound primarily intronic sequences, and RBFOX2 and TDP-43 interacted with intronic sequences and 3’UTRs (Fig. 2b). Unsurprisingly, the paralogs NOVA1 and NOVA2 shared a substantial fraction of binding sites, where ~93% of NOVA2 binding sites are also bound by NOVA1 (Fig. 2c, top). Intriguingly, > 10% of RBFOX2 sites were shared with TDP-43 (Fig. 2c, top). At the gene transcript level, ~60% of TDP-43 targets were also NOVA1 targets (Fig. 2c, bottom). K-mer analysis retrieved reproducible, known, and highly enriched sequence motifs for TDP-43 (UG-rich; Fig. 2d) [52, 64], NOVA (YCAY; Fig. 2e and f) [65] and RBFOX2 (GCAUG; Fig. 2g) [15, 72], as expected. K-mer analysis of RBFOX2 binding sites also revealed enrichment of UG-rich sequences, as observed in previous studies [15, 72] (Fig. 2g). Thus, eCLIP analysis of NOVA1, NOVA2, RBFOX2 and TDP-43 in iPSC-MNs robustly revealed their known binding preferences at the gene region and sequence motif levels.

To associate the binding sites of these RBPs with AS changes, we generated corresponding paired-end RNA-seq datasets (> 37 million reads in each sample). We focused on the most frequent type of AS, cassette exon inclusion and exclusion. We also included identification of novel, unannotated exon junctions to spot potential cryptic splicing events. We focused on exon-exon spanning junction reads, and compared the ALS datasets with the Ctrl (Online Resource Fig. 3a). We identified 2,806 differentially spliced cassette exons in our sALS vs Ctrl comparison and 4,898 in the fALS comparison that satisfied our stringency thresholds (FDR < 0.05; |Inclusion Level Difference| > 0.1; coverage > 10 reads in each sample per event). Familial and sporadic ALS exhibited a significant overlap and correlation in AS changes (Online Resource Fig. 3d–f). Focusing our analysis on the pre-mRNA region encompassed by the upstream exon and the downstream exon, we observed that NOVA1, NOVA2, and RBFOX2 binding sites were statistically enriched (in both eCLIP-seq replicates) at cassette exons alternatively spliced between sALS (Fig. 2h) and fALS (Fig. 2i) relative to Ctrl. The statistical significance of enrichment of these three proteins was consistently higher compared to TDP-43 (Fig. 2h and i). To independently validate these findings, we included RNA-seq data of iPSC-derived MNs from ALS patients and controls from the AnswerALS consortium (https://www.answerals.org/). AS differences were determined from sALS iPSC-MN and Ctrl (sALS-AA) and C9ORF72 hexanucleotide repeat expansion (C9-ALS-AA) iPSC-MN (Online Resource Fig. 3a and b). We found that AS changes were also prominent in these two independent ALS datasets (sALS-AA: 1,001 significant AS events; C9-ALS-AA: 649 significant AS events; Online Resource Fig. 2c-f). Strikingly, similar to our own datasets, NOVA1, NOVA2 and RBFOX2 binding sites were also statistically enriched at cassette exons alternatively spliced between ALS and Ctrl in these datasets from the AnswerALS consortium (Fig. 2j and k). To investigate the specificity of this finding, we included a cohort of hereditary spastic paraplegia (HSP) patients carrying pathogenic variants in SPG11 (SPG11-HSP). SPG11-HSP is also a motor neuron disorder that is primarily characterized by upper motor neuron loss and thinning of the corpus callosum. More importantly, SPG11-HSP patients also suffer from amyotrophy, a sign of lower motor neuron degeneration [59]. Despite this strong clinical overlap with ALS, SPG11-HSP forms a disease entity that is distinct from ALS. Hence, SPG11-HSP-associated AS can serve as a non-ALS-related control. We have previously characterized iPSC-MN from SPG11-HSP patients and identified an axon-specific mitochondrial pathology [25]. Now, we performed RNA-seq followed by AS analysis and identified 277 significant AS events in SPG11-HSP (Online Resource Fig. 4). RBFOX2 binding was also robustly enriched in AS in SPG11-HSP in one of the two eCLIP replicates (Fig. 2l). NOVA1 and NOVA2 were ~1.5 fold enriched in SPG11-HSP. However, the degree of significance of enrichment was at least ~10^5^ fold lower in SPG11-HSP compared to the ALS datasets (Fig. 2l). We conclude that NOVA1, NOVA2, and RBFOX2 binding is strongly correlated with AS events that are differentially regulated in ALS, and that NOVA1 and NOVA2 exhibit a robust enrichment especially in ALS.

**Fig. 3:**
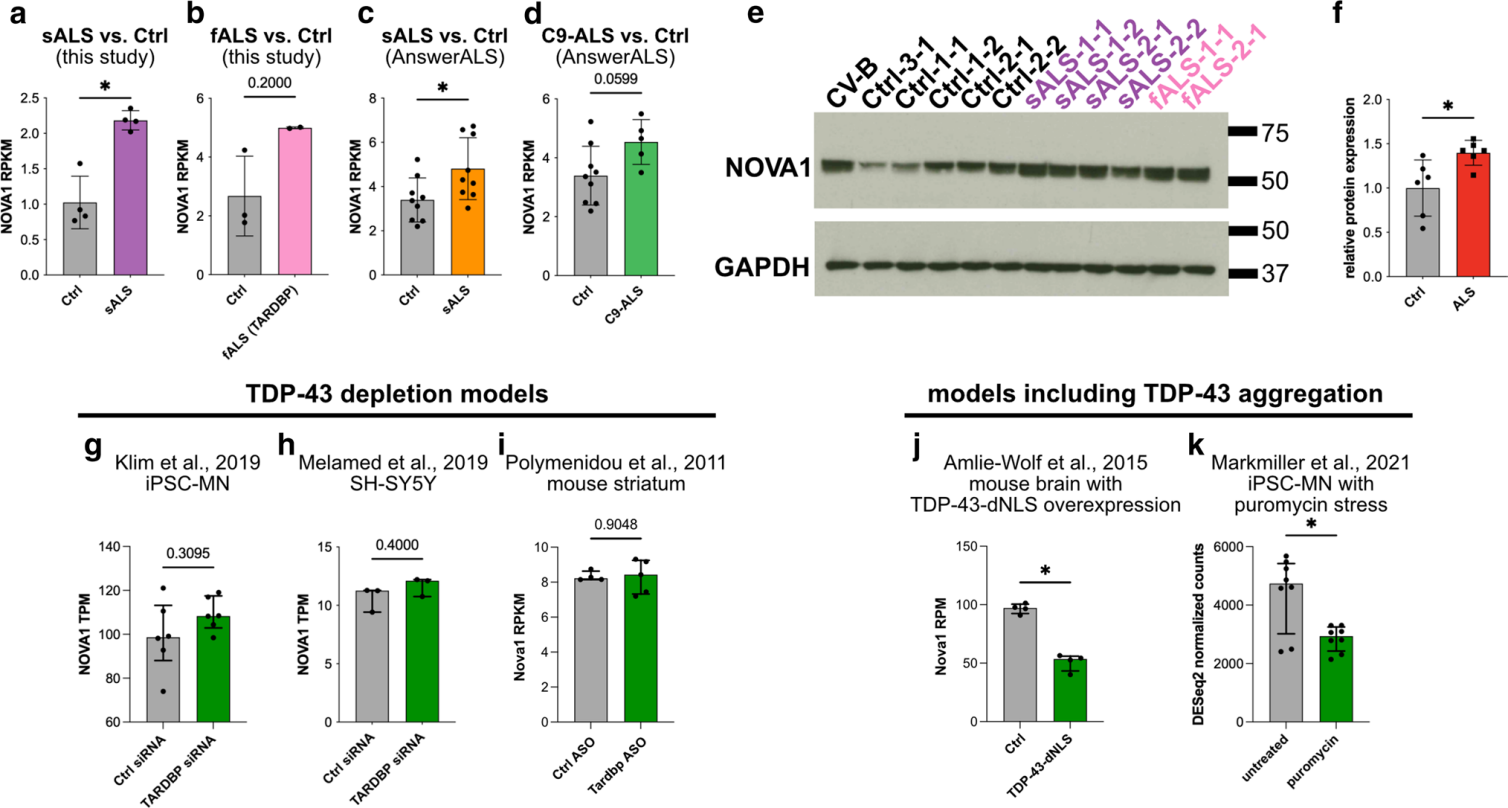
NOVA1 expression is increased in ALS iPSC-MN and elevated cytoplasmic TDP-43 levels are associated with a reduction of NOVA1 mRNA levels. **a**–**d** Bar graphs of gene expression values for NOVA1 as reads per kilobase transcript per million mapped reads (RPKM) from **a** sALS and Ctrl generated in this study (*n *= 4 sALS, *n *= 4 Ctrl), **b** fALS and Ctrl generated in this study (*n *= 2 fALS, *n *= 3 Ctrl), **c** sALS and Ctrl from the AnswerALS consortium dataset (*n* = 9 sALS, *n* = 9 Ctrl), and **d** C9-ALS and Ctrl from the AnswerALS consortium dataset (*n* = 5 C9-ALS, *n* = 9 Ctrl). Data presented as mean ±SD. Statistical significance was determined by Mann-Whitney *U* test. **a** *P* value = 0.0286, **b** *P* value = 0.2000, **c** *P* value = 0.0315, **d** *P* value = 0.0599. **e** Western blot from total cell lysates of iPSC-MNs stained for NOVA1 and GAPDH. **f** Bar graph showing densitometric quantification of Western blot of NOVA1 normalized to GAPDH (*n *= 6 ALS; *n* = 6 Ctrl). Fold change ALS/Ctrl: 1.42. Data presented as mean ±SD. Statistical significance was determined by Mann-Whitney U test. *P* value = 0.0152. **g** Bar plot with NOVA1 expression values (in TPM) in iPSC-MN with control siRNA or siRNA targeting TARDBP (GEO ID: GSE121569; *n* = 6 control siRNA, *n* = 6 TARDBP siRNA). Data presented as median +IQR. Statistical significance was determined by Mann-Whitney *U* test. *P* value = 0.3095. **h** Bar plot with NOVA1 expression values (in TPM) in SH-SY5Y with control siRNA or siRNA targeting TARDBP (GEO ID: GSE122069; *n *= 3 control siRNA, *n *= 3 TARDBP siRNA). Data presented as median +IQR. Statistical significance was determined by Mann-Whitney *U* test. *P* value = 0.4000. **i** Bar plot with NOVA1 expression values (in RPKM) in mouse striatum with control ASO or ASO targeting TARDBP (GEO ID: GSE27394; *n* = 4 control ASO, *n *= 5 TARDBP ASO). Data presented as median +IQR. Statistical significance was determined by Mann-Whitney *U* test. *P* value = 0.9048. **j** Bar plot with NOVA1 expression values (in RPM) in mouse brain without and with TDP-43-dNLS expression (GEO ID: GSE65973; *n *= 4 Ctrl, *n *= 4 TDP-43-dNLS). Data presented as median +IQR. Statistical significance was determined by Mann-Whitney *U* test. *P* value = 0.0286. **k** Bar plot with NOVA1 expression values (in DESeq2 normalized counts) in iPSC-MN (4 Ctrl, 4 HNRNPA2B1 mutant lines) without and with puromycin stress (GEO ID: GSE157467; *n* = 8 untreated, *n* = 8 puromycin). Data presented as median +IQR. Statistical significance was determined by Mann-Whitney *U* test. *P* value = 0.0379

### Elevation of NOVA1 is observed in ALS iPSC-MN, and reduction of NOVA1 levels is observed upon cytoplasmic TDP-43 localization in mice and chemical stress in iPSC-MN

NOVA proteins are neuronally enriched AS factors. Nova1 knock-out (KO) in mice is postnatally lethal due to progressive motor dysfunction with increased cell death in the spinal cord [28]. Expression analyses in mice suggest a reciprocal expression pattern for Nova1 and Nova2, where Nova2 is predominantly expressed in the cortex and hippocampus, while higher Nova1 levels are observed in midbrain and spinal cord [56]. This strong link between Nova1 and spinal motor neuron biology led us to focus on the association of NOVA1 in human MNs and alterations of its function in ALS.

Interestingly, NOVA1 mRNA levels were significantly increased in sALS iPSC-MN (Fig. 3a) and a trend (*P* = 0.2) of increased NOVA1 mRNA levels was also evident for the fALS samples (Fig. 3b). An increase in NOVA1 levels was also observed in the sALS-AA dataset (Fig. 3c) while the increased trend in C9-ALS-AA did not reach statistical significance (*P* value = 0.0599; Fig. 3d). This increase of NOVA1 mRNA in ALS was also reflected by protein levels (Fig. 3e and f). Next, we asked if TDP-43 function, specifically loss of function or cytoplasmic toxic gain of function, is associated with altered NOVA1 mRNA levels. We analyzed 3 frequently used TDP-43 depletion RNA-seq datasets [31, 46, 52] and did not identify changes in NOVA1 mRNA levels (Fig. 3g–i). However, a significant reduction of NOVA1 mRNA level was detected in a model where TDP-43 lacking its nuclear localization signal was overexpressed in mice [3] (Fig. 3j). Application of the chemical stressor puromycin in iPSC-MN, that inhibits protein translation and also facilitates cytoplasmic TDP-43 aggregation, leads to a reduction of NOVA1 levels, too [41] (Fig. 3k). We conclude that NOVA1 levels are increased in iPSC-MN models of ALS and may be decreased in models where TDP-43 is aggregated.

### ALS iPSC-derived motor neurons exhibit disrupted NOVA1 function

**Fig. 4:**
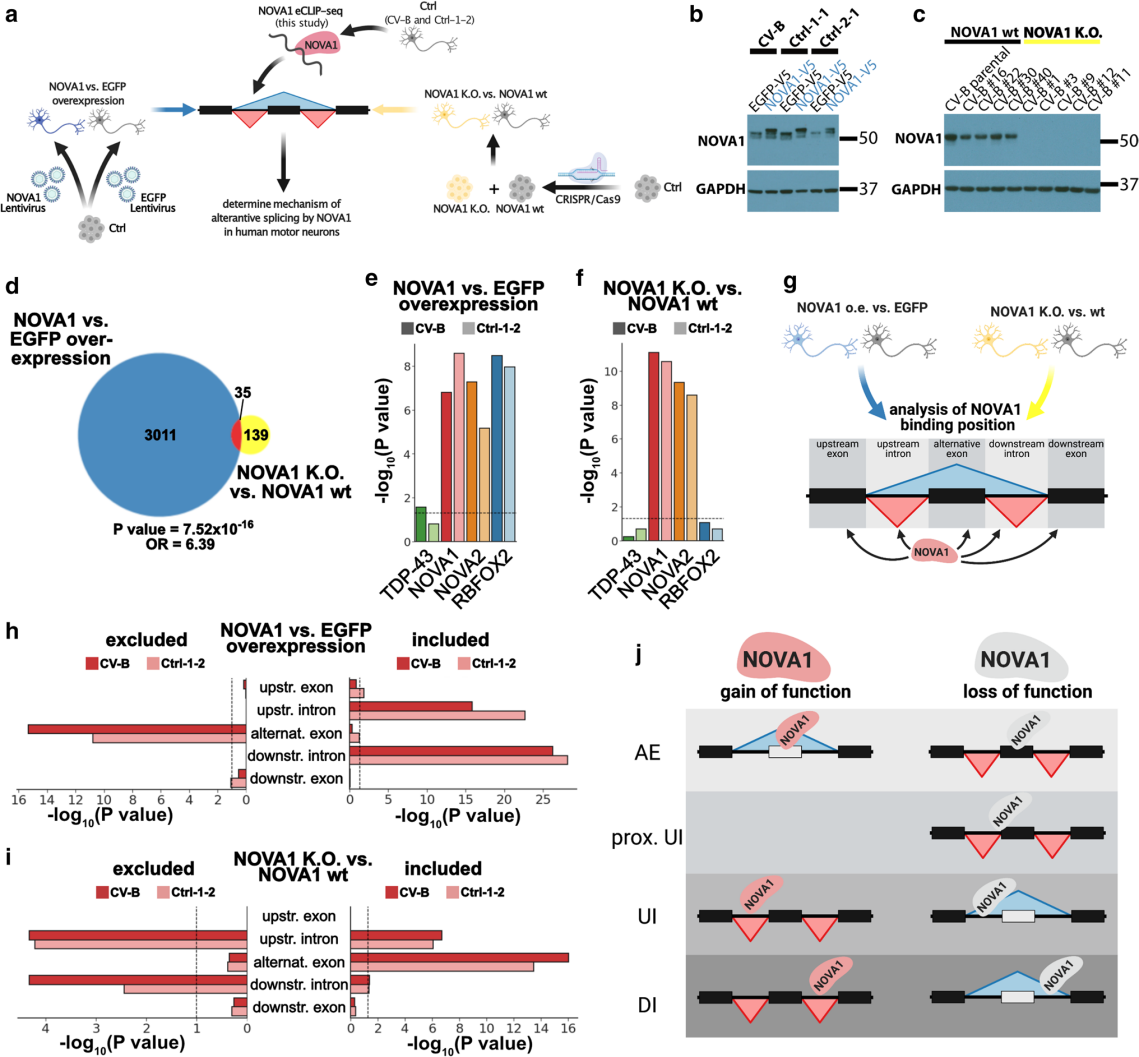
NOVA1 mediates AS in human iPSC motor neurons in a position-dependent manner. **a** Schematic illustrating the experimental workflow. **b** Western blot of NOVA1 from day 30 iPSC-MN after transduction with lentivirus containing EGFP (*n* = 3) or NOVA1 (*n* = 3) ORF expressed under the CAG promoter. GAPDH was used as a loading control. Average fold change of 1.97 (CV-B: 2.2-fold; Ctrl-1-1: 1.6-fold; Ctrl-2-1: 2.1-fold). **c** Western blot of NOVA1 from day 30 control (*n* = 5) and NOVA1 K.O. (*n* = 5) iPSC-MN. GAPDH was used as a loading control. **d** Venn diagram of significant differential cassette exon alternative splicing events from comparisons of NOVA1 vs. EGFP overexpression (blue) and of NOVA1 wt vs. NOVA1 K.O. (yellow). Significance of overlap was calculated with Fisher's exact test using all events detected in both analyses as background. *OR* = odds ratio. **e**, **f** Bar graphs of significance of differential enrichment of eCLIP-seq binding sites at AS events of TDP-43 (green), NOVA1 (red), NOVA2 (orange) and RBFOX2 (blue) in **e** NOVA1 vs EGFP overexpression, and **f** NOVA1 K.O. vs Ctrl. Experiments were performed in two cell lines (CV-B, dark shading; Ctrl-1-2, light shading). Dashed horizontal line at *P* value = 0.05. Enrichment was calculated using hypergeometric test with all detected events as the background. **g** Schematic illustrating analysis of positions of interest for NOVA1 binding analysis. **h**-**i** Bar graphs of significance of differential enrichment of NOVA1 eCLIP-seq binding sites at excluded (left) and included (right) AS events (upstream exons, upstream introns, alternative exons, downstream introns and downstream exons) in **h** NOVA1 vs EGFP overexpression, and **i** NOVA1 K.O. vs Ctrl. Experiments were performed in two cell lines (CV-B, dark shading; Ctrl-1-2, light shading). Dashed vertical line at *P* value =0.05. Enrichment was calculated using hypergeometric test with all detected events as the background. **j** Model illustrating physiological modes of action of NOVA1 in human iPSC-derived MN. *AE* alternative exon, *UI* upstream intron, *DI* downstream intron

To understand how NOVA1 affects AS in MN, we generated NOVA1 K.O. and NOVA1 over-expressing models (Fig. 4a). For over-expression, we differentiated iPSC to MN and transduced control (wt) iPSC-MNs with lentivirally packaged NOVA1 or EGFP (control) open reading frames at day 22 and day 26 of differentiation, a time point when the majority of cells already reached a postmitotic stage (Fig. 4a). Interestingly, increasing NOVA1 levels in Ctrl iPSC-MN resulted in an elevation of the soluble but not insoluble NOVA1 pool (Online Resource Fig. 5a-c), in contrast to our observations in ALS iPSC-MN (Fig. 1f). To model a loss of function of NOVA1, we knocked out NOVA1 in a Ctrl iPSC line using CRISPR/Cas9 (Fig. 4c, Online Resource Fig. 5d-f). We generated RNA-seq datasets, and performed pair-wise comparisons of differential AS events (NOVA1 over-expression vs. EGFP over-expression: *n *= 3 biological replicates per group; NOVA1 K.O., vs. NOVA1 wt: *n *= 5 clonal cell lines per group). We observed that over-expression of NOVA1 led to a sizable number (*n *= 3046) of AS events, whereas depletion of NOVA1 resulted in fewer AS changes (*n *= 174), with 35 events being called as significant in both datasets (Fig. 4d). As expected, NOVA1 binding sites were enriched at differential AS events in both over-expression (Fig. 4e) and depletion conditions (Fig. 4f). Since NOVA paralogs share binding sites (Fig. 2c), it is not surprising that NOVA2 binding sites are also enriched at AS sites (Fig. 4e and f). Interestingly, differential AS events upon NOVA1 overexpression were also enriched near RBFOX2 binding sites (Fig. 4e) while binding sites for TDP-43 were not enriched (Fig. 4e and f). To understand the position-dependent effects of NOVA1 on AS, we determined enrichment of NOVA1 binding sites at specific locations around AS events and included the directionality of the AS changes (Fig. 4g). While exons that were excluded upon NOVA1 overexpression exhibited enrichment of NOVA1 binding sites within the alternative exon, exons included upon gain of NOVA1 function were enriched for NOVA1 bound at the upstream and downstream introns (Fig. 4h). We observed an opposite pattern of NOVA1 binding in AS events changed upon NOVA1 loss (Fig. 4i). Additionally, NOVA1 binding at the very proximal part of the upstream intron, encompassing the border region to the alternative exon, was associated with exon exclusion upon loss of NOVA1 (Fig. 4i, Online Resource Fig. 6). In the aggregate, these data suggest that NOVA1 binding to an alternative exon promotes its exclusion and binding to the downstream intron elevates exon inclusion levels. By contrast, binding to an upstream intron may have a distance-dependent effect on the alternative exon, with proximal vs distal binding mediating exclusion or inclusion, respectively (Fig. 4j).

To investigate if the altered solubility and levels of NOVA1 in ALS (Figs. 1 and 3) may affect AS, we compared our RNA-seq datasets from altered NOVA1 function (NOVA1 over-expression and NOVA1 K.O. in iPSC-MNs; Fig. 4) and from ALS patient iPSC-MNs (sALS and fALS, and sALS-AA and C9-ALS-AA). We found a significant overlap (~15%) of AS events changed in the ALS datasets and upon NOVA1 over-expression (Online Resource Fig. 7a-d and i-l). A significant, but smaller overlap, was also observed when compared to AS events that changed upon NOVA1 K.O. (Online Resource Fig. 7e-h and m-p). We also quantified AS events altered upon NOVA1 over-expression or depletion by their percent spliced-in values in the ALS patient datasets. Hierarchical clustering of these datasets largely segregated the fALS and sALS samples from Ctrls (Online Resource Fig. 7q-t).

**Fig. 5:**
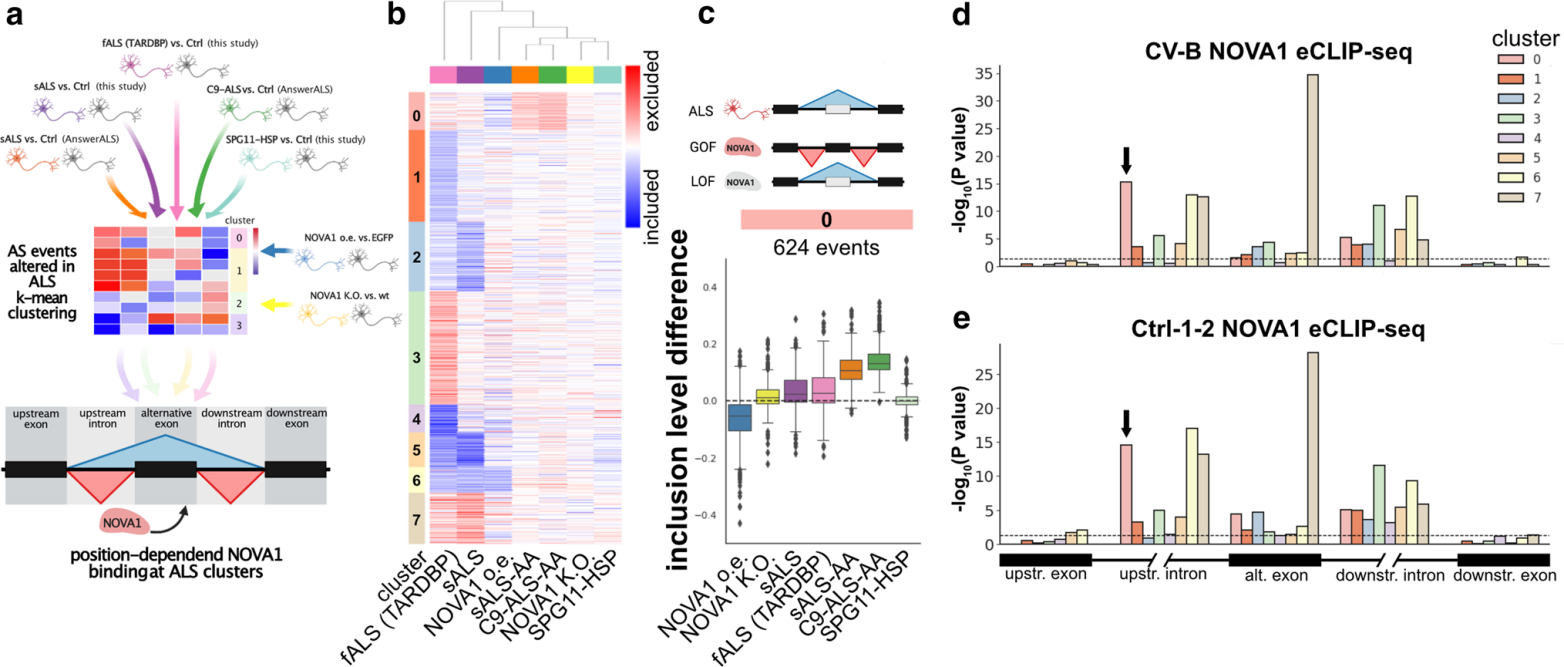
Aberrant NOVA1 states in ALS exhibit gain- and loss-of-function features **a** Schematic illustrating analysis strategy to identify patterns of altered NOVA1 function in ALS iPSC-derived MNs. **b** Heatmap of all significant AS events observed in the four ALS datasets. Inclusion level difference for all depicted datasets were clustered using k-means clustering into 8 distinct clusters. Blue denotes inclusion (max: −0.6), red denotes exclusion (max: 0.6). Datasets are clustered using Euclidean distance metric. **c** Boxplot of inclusion level differences in clusters 0 (sALS vs. Ctrl: purple; fALS vs. Ctrl: pink; sALS vs. Ctrl (AA): orange; C9-ALS vs. Ctrl (AA): green; SPG11-HSP vs. Ctrl: cyan; NOVA1 vs. EGFP overexpression: blue; NOVA1 wt vs. NOVA1 K.O.: yellow). Ideogram on top illustrates the direction of the most prominent observed change in ALS, NOVA1 gain of function (GOF) and NOVA1 loss of function (LOF) (from top to bottom). **d** Bar blot illustrating significance (negative log_10_ scale) of CV-B NOVA1 eCLIP-seq binding site enrichment at AS events in the 8 detected AS event clusters at upstream exon, upstream intron, alternative exon, downstream intron, and downstream exon. Statistical significance was calculated with hypergeometric test against all detected AS events in the four ALS datasets as a background. Dashed line represented *P* value = 0.05. Arrow depicts most significant enrichment in cluster 0. **e** Bar blot of significance (negative log_10_ scale) of Ctrl-1-2 NOVA1 eCLIP-seq binding site enrichment at AS events in the 8 detected AS event clusters at upstream exon, upstream intron, alternative exon, downstream intron and downstream exon. Statistical significance was calculated with hypergeometric test against all detected AS events in the four ALS datasets as a background. Dashed line represents *P* value = 0.05. Arrow depicts most significant enrichment in cluster 0

To identify NOVA1’s exact mode of action in ALS, we employed unsupervised k-means clustering, using the inclusion level differences of 7,282 AS events significantly changed AS events in at least one of the 4 ALS datasets. Subsequently, we applied the two NOVA1 datasets and the complementary SPG11-HSP dataset. Additionally, we integrated information gained from the analysis of position-dependent AS of NOVA1 (Fig. 5a). We computed 8 clusters (0-7) grouping AS events that are changed in at least one of the 4 ALS comparisons (Fig. 5b). Five clusters (1, 2, 3, 4, and 7) had strong ALS dataset specificity, and AS events showed moderate or no changes upon alteration in NOVA1 levels (Online Resource Fig. 8). Clusters 5 and 6 diverged between the four ALS datasets and showed a reciprocal directionality between the two NOVA1 datasets (Online Resource Fig. 8). Interestingly, cluster 0 exhibited exon exclusion in all four ALS datasets and in NOVA1 K.O. conditions, but inclusion in NOVA1 over-expressing iPSC-MN (Fig. 5c). SPG11-HSP patients did not exhibit a change in cluster 0 AS events (Fig. 5c). Integrating our eCLIP-seq data, we detected low significance in NOVA1 binding enrichment in clusters 1, 2, 3, 4, and 5 (Fig. 5d and e). Cluster 7 exhibited strong binding to the alternative exon (Fig. 5d and e, brown bar), yet changes upon altered NOVA1 levels were moderate, indicating that these events may be influenced by NOVA1 but cannot be changed by only altering NOVA1 levels (Online Resource Fig. 8). The changes in cluster 6 were accompanied with a strong enrichment of binding of NOVA1 to upstream intron (Fig. 5d and e, yellow bar), indicating that this cluster is NOVA1 influenced with mixed, patient-specific patterns of directionality. Interestingly, cluster 0 exhibited a strong association of NOVA1 binding to the upstream intron (Fig. 5d and e, salmon color bar, arrow). Taken together with the observed changes in AS (Fig. 5c), cluster 0 represents an ALS-specific NOVA1 loss-of-function signature. Concludingly, in this early stage model of ALS, increased insolubility of NOVA1 (Fig. 1) and elevation of NOVA1 levels (Fig. 3) are accompanied most consistently by a disease-specific ALS AS cluster associated with NOVA1 loss of function and multiple AS events with mixed patterns of altered NOVA1 function.

### NOVA1 expression changes dynamically depending on the disease state in ALS postmortem tissue MN

iPSC-derived models represent an early stage of disease. To investigate histopathological alterations of NOVA1’s cellular expression patterns in early stages of ALS, we conducted a neuropathological analysis of *postmortem* spinal cord sections. Since we are especially interested in MN at an early stage of disease, we employed knowledge from neuropathological observations for their identification. TDP-43 pathology spreads in a sequential pattern throughout the CNS. Neurons in ALS patients that do not exhibit TDP-43 pathology are at an earlier stage of disease. Neurons that do exhibit TDP-43 pathology are already at a propagated, late stage of disease [9]. Hence, individual MNs within an ALS patient can be categorized into two groups: early stage MNs (adequate nuclear TDP-43 expression; no cytoplasmic TDP-43 aggregation) and late stage MNs (lost nuclear TDP-43; cytoplasmic TDP-43 inclusions). For the *postmortem* validation we chose to analyze lumbar spinal cord sections of bulbar / upper limb onset ALS patients, ensuring detection of a sufficient amount of MNs with and without TDP-43 pathology [32].

**Fig. 6:**
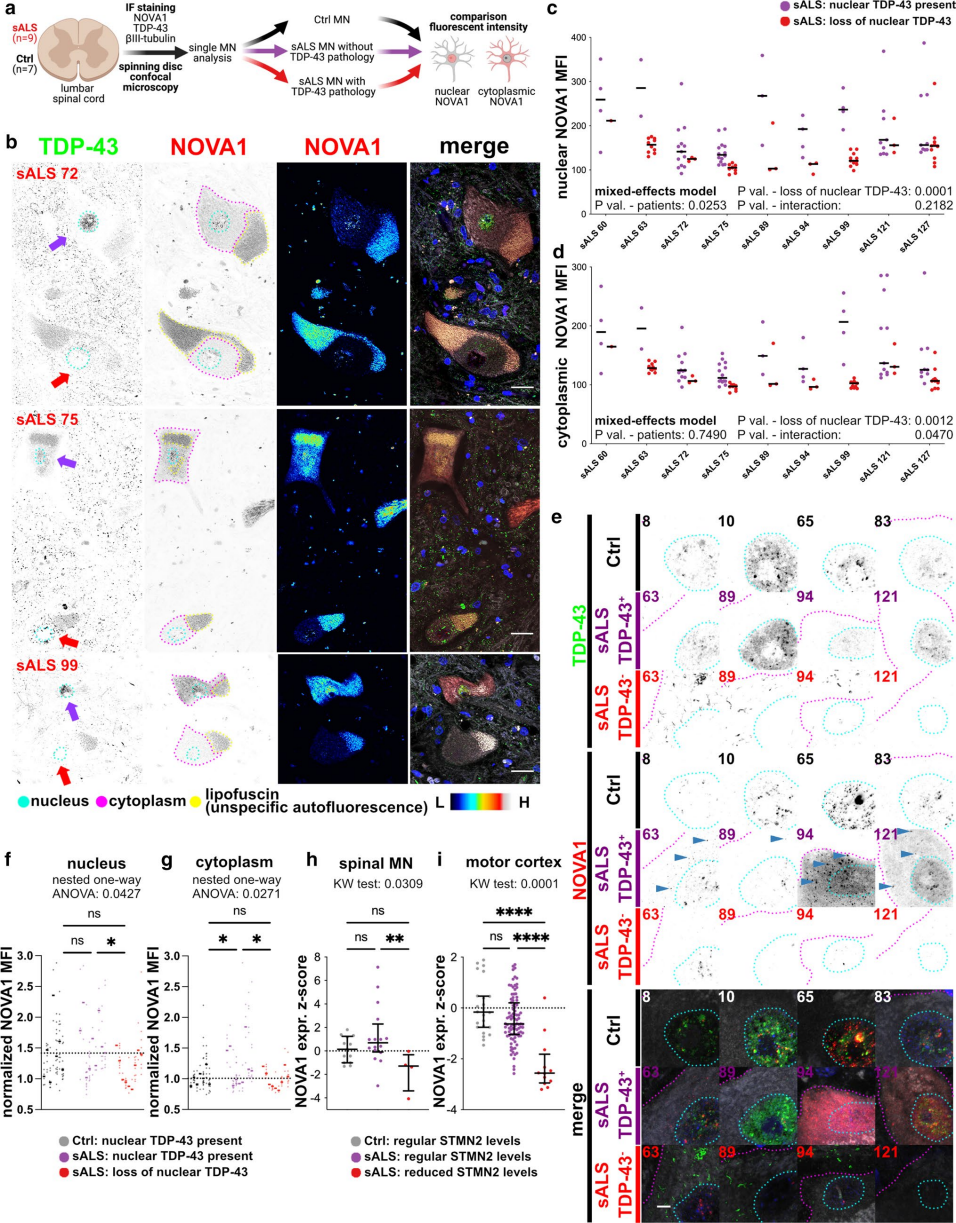
NOVA1 expression in *postmortem* MN in lumbar spinal cord from ALS and Ctrl **a** Schematic illustrating the basic analysis strategy for NOVA1 expression in human tissue. We performed IF staining, spinning disc confocal microscopy and subsequent analysis of single MNs for NOVA1 mean fluorescent intensity and TDP-43 pathology in *n* = 7 Ctrl and *n* = 9 sALS. **b** Representative images of MN with nuclear TDP-43 (no TDP-43 pathology, purple arrow) and without nuclear TDP-43 (with TDP-43 pathology, red arrow) in sALS patients. Pictures stained for TDP-43 (green), NOVA1 (red), bIII-tubulin (gray) and DAPI (blue). Nuclear (cyan), cytoplasmic (fuchsia) and lipofuscin (yellow) areas analyzed are surrounded. Scale bars: 25µm. **c** Scatter plot of mean nuclear NOVA1 staining intensities in individual MNs from different ALS patients without (purple) and with (red) TDP-43 pathology. Horizontal black lines represent medians. Statistical analysis was performed using a mixed effects model. **d** Scatter plot of mean cytoplasmic NOVA1 intensity in individual MNs from different ALS patients without (purple) and with (red) TDP-43 pathology. Horizontal black lines represent medians. Statistical analysis was performed using a mixed effects model. **e** Representative immunofluorescence images of MNs in lumbar spinal cord from control individuals (black) and from sALS patients without (purple) and with TDP-43 pathology (red). Sections were stained for TDP-43 (green), NOVA1 (red), bIII-tubulin (gray) and DAPI (blue). Nuclear (cyan) and cytoplasmic (fuchsia) areas analyzed are surrounded. Blue arrowheads indicate cytoplasmic NOVA1 accumulations. Scale bar: 25µm. **f** Scatter plot of bIII-tubulin normalized mean nuclear NOVA1 staining intensities of single MNs (dots) their average in an individual (line). Dashed line represents median in Ctrl. Statistical significance was determined by nested one-way ANOVA. *P value < 0.05; **P value < 0.01; ***P value < 0.001; ****P value < 0.0001. Significant variation between individuals within a group: *P* value = 0.0001. **g** Scatter plot of bIII-tubulin normalized mean cytoplasmic NOVA1 intensities of single MN (dots) their average in an individual (line). Dashed line represents median in Ctrl. Statistical significance was determined by nested one-way ANOVA. *P value < 0.05; **P value < 0.01; ***P value < 0.001; ****P value < 0.0001. Significant variation between individuals within a group: *P* value > 0.05. **h** Scatter plot illustrating NOVA1 expression converted into expression Z-scores based on control means from *postmortem* ALS LCM datasets from [32] and [55] in Ctrl (black), sALS samples with regular STMN2 levels (STMN2 expression Z-score > −2, purple) and sALS samples with reduced STMN2 levels (STMN2 expression Z-score < −2, red). Black horizontal lines indicate median and interquartile ranges. Statistical significance was determined by Kruskal-Wallis test and Dunn's post hoc test to identify changes between individual groups. *P value < 0.05; **P value < 0.01; ***P value < 0.001; ****P value < 0.0001 **i** Scatter plot illustrating NOVA1 expression converted into expression Z-scores based on Ctrl means from *postmortem* ALS motor cortex datasets from UCSD and NYGC cohorts [63] in Ctrl samples (black), sALS samples with regular STMN2 levels (STMN2 expression Z-score > −2, purple), and sALS samples with reduced STMN2 levels (STMN2 expression Z-score < −2, red). Black horizontal lines illustrate median and interquartile ranges. Statistical significance was determined by Kruskal-Wallis test and Dunn's post hoc test to identify changes between individual groups. *P value < 0.05; **P value < 0.01; ***P value < 0.001; ****P value < 0.0001

We classified TDP-43 pathology in MN of spinal cord sections from 9 sALS cases and 7 Ctrls (including 2 Alzheimer’s disease patients) followed by determination of NOVA1 expression patterns (Fig. 6a, Online Resource Figure 9, Online Resource Figure 10a, and Online Resource Table 3). The amount of MNs with loss of nuclear TDP-43 was variable in sALS (Online Resource Fig. 10b). Nuclear loss of TDP-43 was not observed in Ctrl MNs (Online Resource Fig. 10b). Interestingly, the vast majority of sALS MNs, with nuclear TDP-43 retention did not exhibit cytoplasmic TDP-43 accumulation (Online Resource Fig. 10b). Hence, for further analysis we only considered the nuclear presence of TDP-43 (Fig. 6b, purple arrow, early disease stage MN) or absence of nuclear TDP-43 (Fig. 6b, red arrow, late disease stage MN) as a metric segregating sALS MNs into two categories. First, we compared basic morphological parameter. We found that the soma size (Online Resource Fig. 10c), nuclear size (Online Resource Fig. 10d) and the area of lipofuscin (Online Resource Fig. 10e) in MNs were comparable between the groups.

Significantly lower levels of nuclear NOVA1 were present in MNs with loss of nuclear TDP-43 (Fig. 6c). In contrast, MNs where nuclear TDP-43 was present appeared to have a significant increase in cytoplasmic NOVA1 (Fig. 6d). To investigate how these changes compare to physiological expression patterns, we also analyzed MNs from Ctrl samples (Fig. 6e). To address the typically high level of variability of *postmortem* analyses, we normalized individual NOVA1 fluorescence intensities in MNs to their respective bIII-tubulin fluorescence intensities (Online Resource Fig. 10f), thereby successfully reducing the inter-individual variance in Ctrls by ~50% for nuclear NOVA1 signal and ~75% for cytoplasmic NOVA1 signal (Online Resource Fig. 10g). To validate our analysis approach, we also investigated the TDP-43 fluorescent intensities. As expected, nuclear TDP-43 levels are decreased when TDP-43 is lost from the nucleus (Online Resource Fig. 10h) and cytoplasmic levels are increased when nuclear TDP-43 is lost (Online Resource Fig. 10i). Regarding NOVA1 expression, sALS MNs with retained nuclear TDP-43 showed a regular nuclear NOVA1 expression (Fig. 6f). Strikingly, sALS MNs without nuclear loss of TDP-43 exhibited increased cytoplasmic NOVA1 signal intensities (Fig. 6g). In turn, sALS MNs with loss of nuclear TDP-43 had lower nuclear NOVA1 signal than Ctrls (Fig. 6f). We also investigated the cellular localization of NOVA1 in iPSC-MN and found that higher cytoplasmic NOVA1 levels are also observed in some but not all of the analyzed patients (Online Resource Fig. 11)

To validate the changing pattern of NOVA1 expression independently, we used publicly available ALS *postmortem* RNA-seq datasets from laser captured MNs [32, 55] and motor cortex [63]. These RNA-seq datasets represent a pool of MNs with nuclear loss of TDP-43 and MNs with retained nuclear TDP-43 expression. It was previously proposed that STMN2 levels can be a proxy for nuclear TDP-43 function, where STMN2 levels decrease with loss of nuclear TDP-43 function [31, 46]. Therefore, we tested whether changes in NOVA1 expression are correlated with a reduction of STMN2 levels (and thus reduced nuclear TDP-43). Interestingly, we found that NOVA1 expression exhibited a significant positive correlation with STMN2 mRNA levels in sALS samples, while no significant correlation was observed in Ctrls in all four datasets (Online Resource Fig. 12a-d). We then separated the sALS patients in STMN2 high (normal TDP-43 function) and STMN low expressers (reduced TDP-43 function) (Online Resource Fig. 12e and f). We observed a significant decrease in NOVA1 expression in motor cortex samples with low STMN2 expression (Fig. 6i) and a similar trend in the laser captured MN datasets from sALS (*P* value = 0.0584) (Fig. 6h), in line with our observations that NOVA1 levels are reduced upon TDP-43 expression lacking its nuclear localization signal and application of a chemical stressor (Fig. 3j and k). We conclude that in early stages of ALS, cellular NOVA1 levels are increased, in particular in the cytoplasm. In later stages, NOVA1 levels are reduced once TDP-43 is cytoplasmically aggregated and the onset of pathology has begun.

## Discussion

**Fig. 7:**
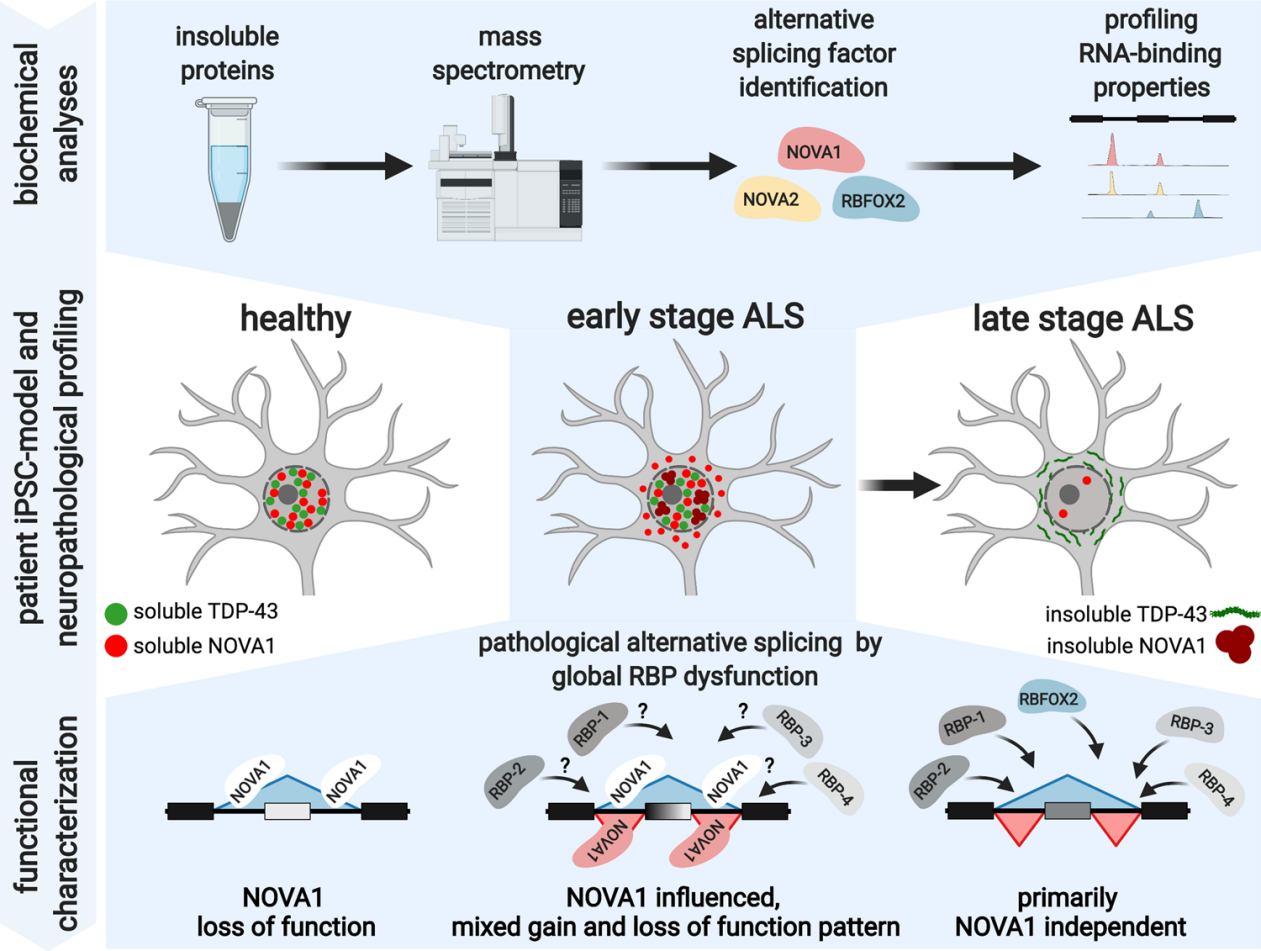
Paradigm of proposed NOVA1 pathological changes and functional aberrations during early and late stages of ALS disease course as determined by TDP-43 pathology. Altered cellular and functional properties of NOVA1 are specifically present at early stages of ALS

In this study, we explored the perturbation of RBP-splicing networks in early stages of ALS. Using label-free mass spectrometry, we identified 88 candidate proteins enriched in the insoluble fraction of ALS patient-derived iPSC-MN, among the RBPs NOVA1, NOVA2, ELAVL4, RBFOX2 and RBFOX3. Using eCLIP analysis, we identified enriched binding of NOVA1, NOVA2 and RBFOX2 at alternatively spliced cassette exons in ALS. In contrast, NOVA proteins were less significantly enriched at AS sites in a non-ALS motor neuron disease cohort. Integrating NOVA1 overexpression and depletion RNA-seq datasets, we provide evidence that NOVA1’s AS function is disrupted in ALS, including a consistent loss of NOVA1 function signature. On a cellular level, we detected an increased expression of the protein in ALS. In *postmortem* spinal cord tissue from ALS patients, the expression pattern of NOVA1 is stage dependent, with elevated cytoplasmic NOVA1 levels already present in MNs that do not yet exhibit TDP-43 pathology. Altered cellular and functional properties of NOVA1 characterize the molecular pathogenesis of ALS preceding the neuropathological hallmark of the disease (Fig. 7).

TDP-43 pathology is considered to lead to a loss of nuclear function and a toxic cytoplasmic gain of function. Interestingly, we identify that loss of TDP-43 function does not alter NOVA1 levels. However, cytoplasmic TDP-43 expression in mice and application of an unspecific chemical stressor that also induced TDP-43 aggregation in iPSC-MN appears to be associated with a decrease in NOVA1. These latter observations are limited by the fact that the first data were produced in mice and the second data used a model where a stress signal with various non-specific side effects was used to induce TDP-43 aggregation. Hence, further studies will need to reconfirm these findings in more comprehensive models of TDP-43 aggregation human iPSC-MN. Interestingly, in sALS MN with cytoplasmic TDP-43 aggregation in *postmortem* tissue we can also identify a decrease in NOVA1. This emphasizes a dynamic expression pattern of NOVA1 during the disease course with high NOVA1 levels in early stages that decrease with cytoplasmic TDP-43 aggregation. Hence, this response may be, in part, regulated by toxic gain of function of TDP-43.

While important deleterious downstream effects of defined alternative splicing events upon loss of nuclear TDP-43 function have been recently described [31, 46], studies specifically focusing on pathological alterations of RBPs upstream of aberrant AS in ALS are limited. One of our insoluble RBP candidates, ELAVL4 (also known as HuD), has been previously shown to accumulate in ALS *postmortem* tissue [16, 17]. Neuropathological investigations on another ELAVL family member, ELAVL3, revealed dot-like cytoplasmic accumulations in ALS, also in patients’ MNs without TDP-43 pathology [17]. Interestingly, ELAVL3 barely did not pass our fold change cutoff in our mass spec protein insolubility analysis (Fig. 1, *P* value = 0.0043; fold change = 1.43). For ALS, few previous studies included specifically MNs without TDP-43 pathology. Hence, the identified ELAVL3-pathologies [17] and our observation that NOVA1 exhibited increased insolubility in this iPSC-MN model of early stage ALS and higher cytoplasmic levels in *postmortem* MNs that do not yet show TDP-43 pathology strengthen the conclusion that abnormal RBPs alterations occur early in the pathogenesis of the disease. While the functional consequences of ELAVL3 mis-localization remain elusive, our findings suggest that this early stage NOVA1 RBP pathology impacts RNA metabolism.

The NOVA family is important for the development of a mature CNS [70] and NOVA1 in particular presents a striking association with MN biology in mice [28, 56]. Nova proteins are thought to mediate AS through enhancing spliceosome assembly by binding YCAY clusters in a position-dependent manner [65]. In a physiological setting, we identified that NOVA1 binding to an alternative exon may cause exon exclusion, while binding in upstream or downstream introns promotes exon inclusion (Fig. 4). This is coherent with previous observations in mice [36, 74] except binding of Nova proteins to upstream introns was shown to promote only exon exclusion [36, 74]. This expansion of the mode of action could be explained by additional binding of NOVA1 further upstream of the alternative exon (>1kb) in our datasets.

We identified that NOVA1 exhibits gain and loss-of-function features in ALS simultaneously, most consistently NOVA1 loss of function, despite increased expression levels. Recent findings suggest that AS factors dynamically change their function within a cell depending on their expression levels, biochemical solubility, and subcellular localization. Application of chemical stressors leading to RBP accumulation changes the RNA-binding profile of RBPs, including TDP-43 [41]. For RBFOX proteins it has been reported that high local concentrations expand its binding to so-called secondary motifs [7]. Additionally, RBFOX proteins exemplify that the biochemical state of a RBP mediates its specific region binding profile [15] leading to a gain of function as an exon inclusion factor when in a high molecular weight fraction [73]. Interestingly, under physiological conditions Nova1 is predominantly absent of this high molecular weight protein fraction in mice [15], coherent with our NOVA1 insolubility analyses. Lastly, a recent study provides evidence that interaction of different AS factors, including RBFOX2, can facilitate complex AS patterns [75]. Hence, our findings that NOVA1, but also other RBPs like RBFOX2, shift into an insoluble state and change their intracellular localization in ALS patients are in line with the observed complex AS pattern in diseased MNs. Future eCLIP studies of NOVA1 and other RBPs in multiple patients are needed resolve the functional change of NOVA1 binding upon the biochemical changes observed in diseased states with base precision.

In conclusion, our study identifies that cellular and biochemical alterations of various RBPs, including NOVA1, disrupt RBP-splicing networks in ALS at early stages of disease in a complex fashion (Fig. 7). As a result, there is a clear need to further decipher how this aberrant AS at early disease stages modulates cellular pathways contributing to MN death. Additionally, independent studies in larger cohorts and different brain areas will be necessary to determine the detailed extend of our observations. However, our study illustrate the complexity of RBP-RNA interactions in ALS. Hence, therapeutic strategies may need to take into account RBP-RNA perturbations in parallel to TDP-43 as early interventions preventing MN death.

## Supplementary Information

Below is the link to the electronic supplementary material.

Supplementary file1 (PDF 2365 kb)

## Data Availability

Data without potential to re-identify individuals (RNA-seq: counts, DESeq2 output, rMATS output; eCLIP-seq: annotated peak file; mass spec: MaxQuant output; FACS: fcs files; Western blot: uncropped images; postmortem image analysis: quantifications of individual MN) is publicly accessible via Mendeley Data (http://dx.doi.org/10.17632/v7p6dh5tvc.1). Due to specific patient consents of individuals who donated biological material to conduct this study, raw data cannot be distributed through an unrestricted access. NOVA1 overexpression, NOVA1 knock-out and in house generated ALS RNA-seq and eCLIP-seq raw data are deposited in the European Genome-Phenome Archive (EGA, ega-archive.org; sALS vs. Ctrl RNA-seq: EGAD00001008424; fALS vs. Ctrl RNA-seq: EGAD00001008425; TDP-43 eCLIP: EGAD00001008426; NOVA1, NOVA2, RBFOX2 eCLIP: EGAD00001008428; EGFP vs. NOVA1 overexpression RNA-seq: EGAD00001008423; NOVA1 wt vs. NOVA1 K.O. RNA-seq: EGAD00001008427). The mass spectrometry proteomics data have been deposited to the ProteomeXchange Consortium via the PRIDE partner repository with the dataset identifier PXD032140. The SPG11-HSP RNA-seq raw data is available through the corresponding author upon reasonable request. Any additional information required to reanalyze the data reported in this paper is available from the lead contact upon request.

## Materials availability

All generated plasmids are available from the lead contact upon reasonable request. There are restrictions to the availability of certain iPSC lines used in this study due to the vote of the ethics committee. A detailed list of all materials, softwares and data are given in Online Resource Table 4.

## Code availability

This study does not report original code.
