# Supplementary material for: Aberrant NOVA1 function disrupts alternative splicing in early stages of amyotrophic lateral sclerosis
Source: Acta Neuropathol. 2022 Jul 1;144(3):413–35. doi: 10.1007/s00401-022-02450-3 (PMC9381448; doi:10.1007/s00401-022-02450-3)
Supplement: Supplementary file 1 — Supplementary file1 (PDF 2365 kb) [file 401_2022_2450_MOESM1_ESM.pdf]

Supplementary Figures and Tables

Aberrant NOVA1 function disrupts alternative splicing in early stages of amyotrophic lateral sclerosis

Florian Krach[1,2], Emily C. Wheeler[2], Martin Regensburger[1,3,4], Tom Boerstler[1], Holger Wend[1], Anthony Q. Vu[2], Ruth Wang[2], Stephanie Reischl[1], Karsten Boldt[6], Ranjan Batra[2], Stefan Aigner[2], John Ravits[5], Juergen Winkler[3,4], Gene W. Yeo[2]*, Beate Winner[1,3]*

[1] Department of Stem Cell Biology, University Hospital Erlangen, Friedrich-Alexander University of Erlangen-Nürnberg (FAU)

[2] Department of Cellular and Molecular Medicine, University of California San Diego

[3] Center of Rare Diseases Erlangen (ZSEER), University Hospital Erlangen, Friedrich-Alexander University of Erlangen-Nürnberg (FAU)

[4] Department of Molecular Neurology, University Hospital Erlangen, Friedrich-Alexander University of Erlangen-Nürnberg (FAU)

[5] Department of Neurosciences, University of California San Diego

[6] Core Facility for Medical Bioanalytics, University of Tübingen, Germany

*co-correspondence: geneyeo@ucsd.edu and beate.winner@fau.de

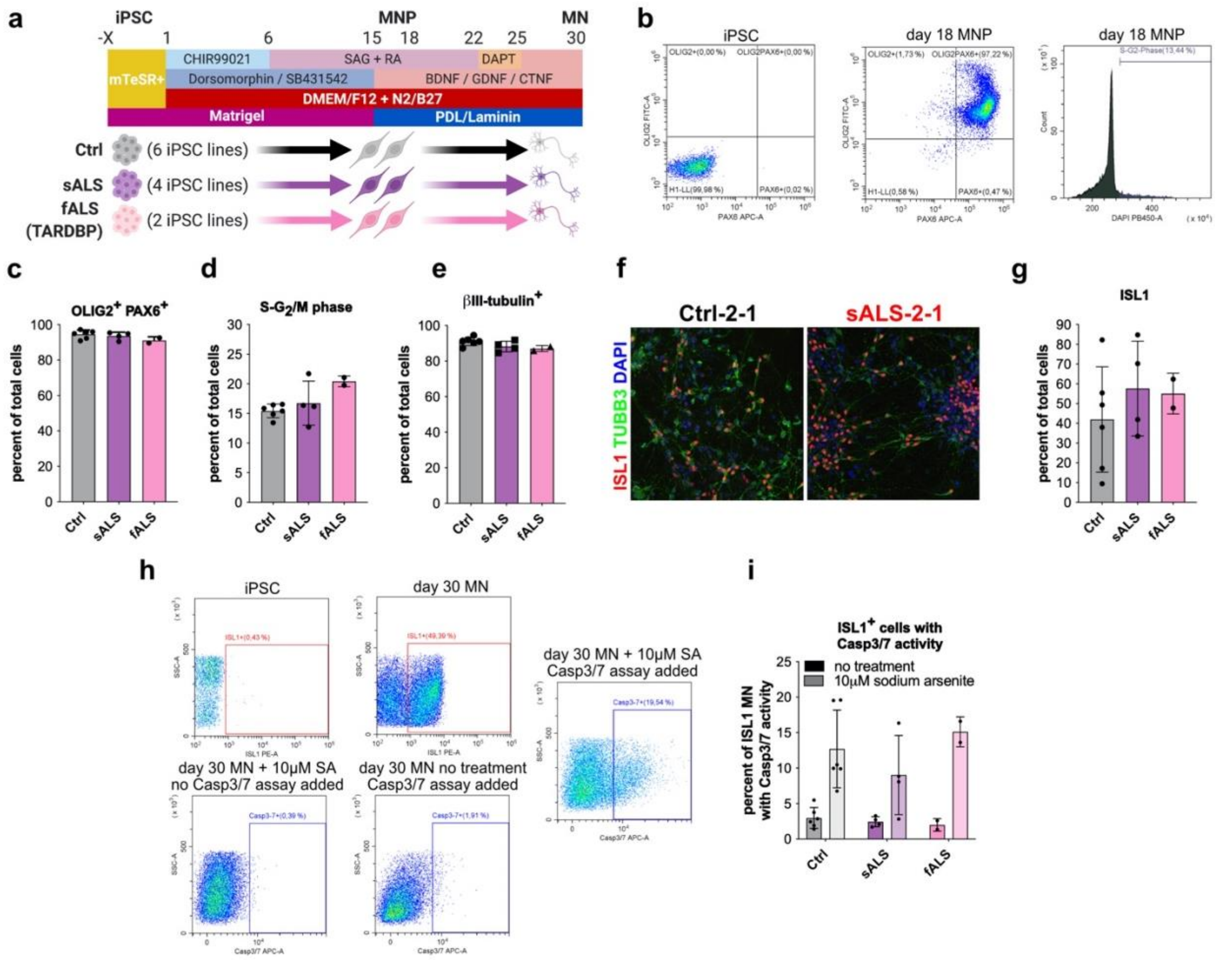

Online Resource Fig. 1. Related to Fig. 1. Differentiation of iPSC into MNs

(a) Schematic showing differentiation protocol with number of lines used from all experimental groups (n=6 ALS, n=6 Ctrl).

(b) gating strategy for OLIG2 / PAX6 FACS with iPSC (left), OLIG2 / PAX6 FACS with d18 MNP (middle) and cell cycle analysis (DAPI; right).

(c) Bar graph of the fraction of OLIG2/PAX6 double positive cells at day 18 of differentiation as measured by FACS. Data presented as mean +SD.

(d) Bar graph of the fraction of cells in S-G2/M phase of cell cycle at day 18 of differentiation as measured by FACS of DAPI stained cells. Data presented as mean +SD.

(e) Bar graph of the fraction of bIII-tubulin positive cells at day 30 of differentiation as measured by FACS. Data presented as mean +SD.

(f) Representative images of MNs at day 30 of differentiation stained for ISL1 (red), bIII-tubulin (green) and DAPI.

(g) Quantification of the fraction of ISL1 positive MNs at day 30 of differentiation. Data presented as mean +SD.

(h) Gating strategy for d30 MN analysis. Top two graphs depict gating for ISL1 in iPSC (left) and MN (right). Bottom two graphs and graph to the total right show gating for Caspase 3/7 assay.

(i) Quantification of the fraction of ISL1 positive MNs with Caspase 3/7 activity after no treatment or treatment with 10µM sodium arsenite for 21h. Data presented as mean +SD.

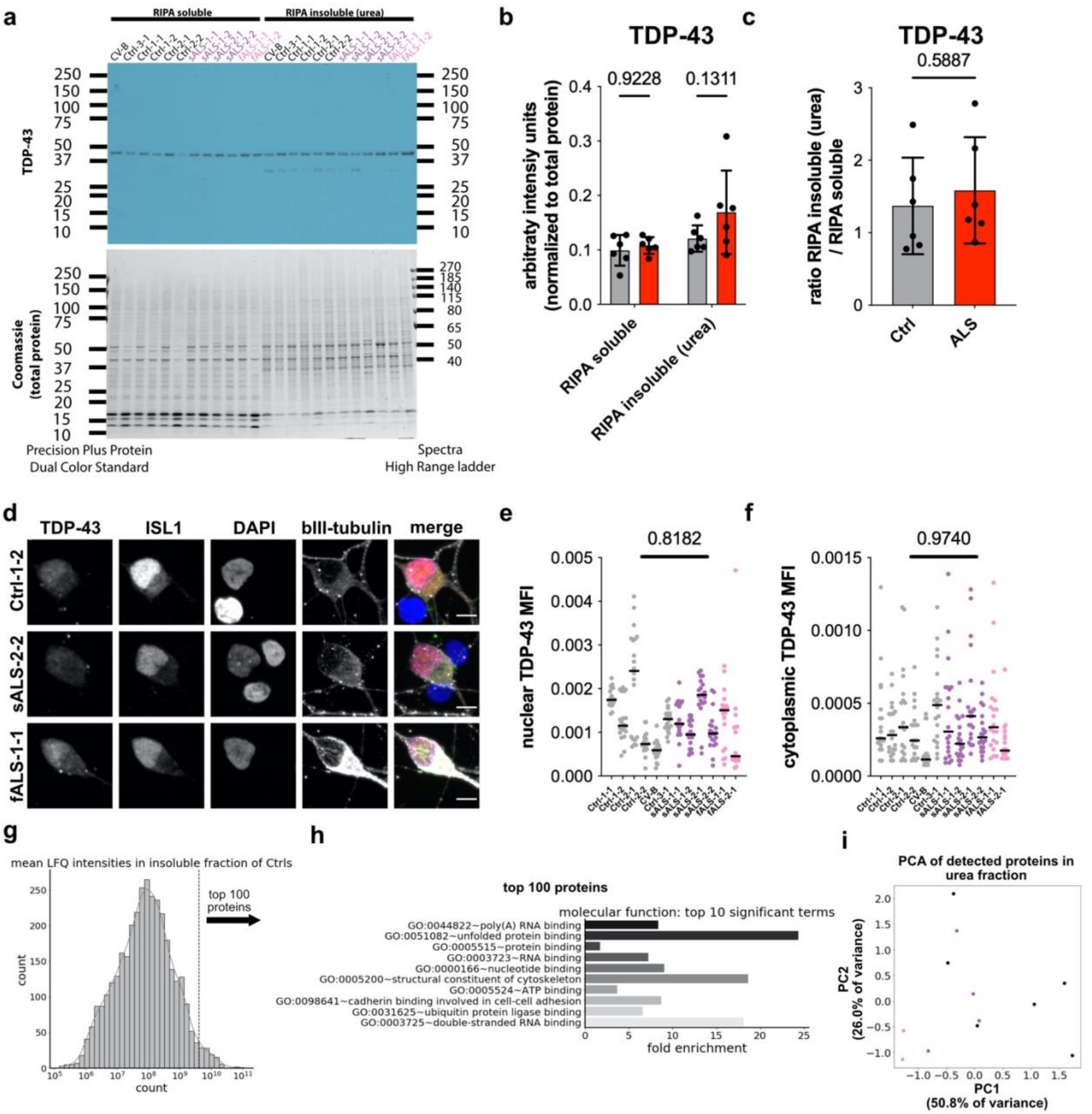

Online Resource Fig. 2. Related to Fig. 1. TDP-43 pathology and protein insolubility analysis

(a) Western blot of TDP-43 in soluble and insoluble fractions and corresponding Coomassie-stained gel (n=6 ALS, n=6 Ctrl).

(b) Densitometric quantification of TDP-43 western blot normalized to the Coomassie controls. Statistical significance was determined by 2-way ANOVA. P values were corrected using FDR. Data presented as mean ±SD (gray bars: Ctrl; red bars: ALS) and individual values (dots) (n=6 ALS, n=6 Ctrl). P value shown as number on the top.

(c) ratio insoluble / soluble of densitometric quantification of TDP-43 western blots normalized to the Coomassie controls. Statistical significance was determined by Mann-Whitney U test. Data presented as mean ±SD (gray bars: Ctrl; red bars: ALS) and individual values (dots) (n=6 ALS, n=6 Ctrl). P value shown as number on the top.

(d) Representative images of iPSC-MNs stained for TDP-43 (green), ISL1 (red), bIII-tubulin (gray) and DAPI (blue). Scale bar: 5µm.

(e) Scatter plot of nuclear TDP-43 mean fluorescent intensities (MFI) of individual ISL1 MN (n=20/cell line; dots) in Ctrl (n=6) and ALS (n=6). Black bar represents median in a cell line. Statistical significance was determined on medians by Mann-Whitney U test. P value = 0.8182.

(f) Scatter plot of cytoplasmic TDP-43 mean fluorescent intensities (MFI) of individual ISL1 MN (n=20/cell line; dots) in Ctrl (n=6) and ALS (n=6). Black bar represents median in a cell line. Statistical significance was determined on medians by Mann-Whitney U test. P value = 0.9740.

(g) Histogram of the mean LFQ intensities of the six control samples resenting values > 0. X-axis in $\log_{10}$ scale.

(h) Bar graph of enrichment of molecular function GO terms of the 100 proteins with the highest LFQ intensities in controls. The top 10 most significantly enriched GO terms are shown.

(i) Principal component analysis (PCA) of LFQ intensities from the different samples (Ctrl: black; sALS: purple; fALS: pink).

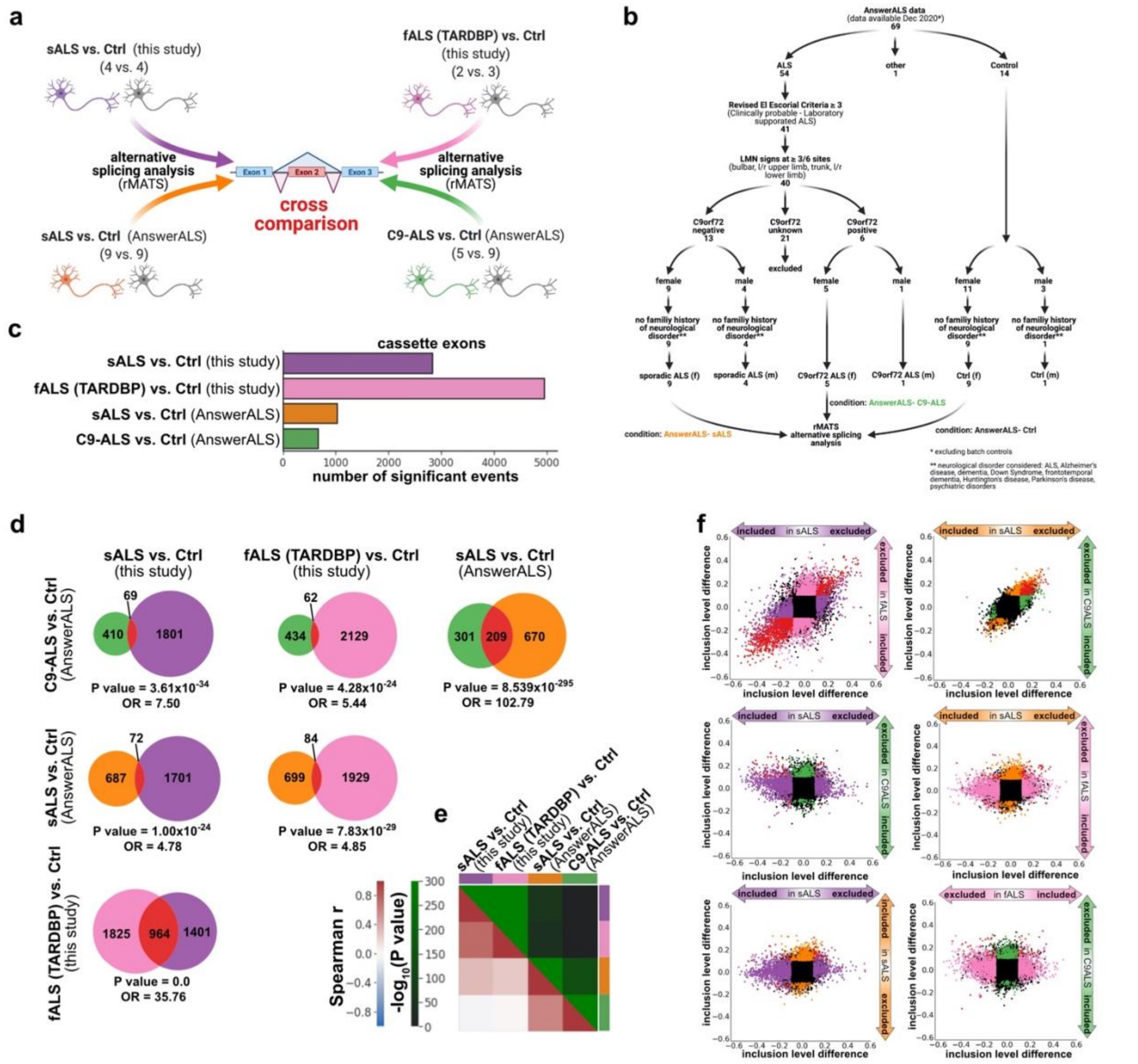

Online Resource Fig. 3. Related to Fig. 2. Degree of AS in ALS iPSC-MNs.

(a) Schematic illustrating the datasets generated in this study (sALS vs. Ctrl, fALS vs. Ctrl), the AnswerALS consortium (sALS vs. Ctrl and C9-ALS vs. Ctrl) and the number of lines included.

(b) Schematic illustrating number of patients and criteria set for inclusion of individuals in our comparisons from the AnswerALS consortium (http://data.answerals.org).

(c) Bar graph of the number of significantly differentially alternatively spliced cassette exons in the four ALS datasets (sALS, fALS, sALS-AA, C9-ALS-AA). FDR < 0.05, |IncLevelDifference| > 0.1, coverage > 10 reads in each sample in a given dataset.

(d) Venn diagrams of pairwise comparisons and overlap of significantly differentially alternatively spliced cassette exons between two datasets. Significance of overlap was calculated with Fisher's exact test using all events detected in both analyses as background. OR = odds ratio.

(e) Heatmap of Spearman correlation coefficients of inclusion level differences from two datasets, respectively. Correlation coefficients are shown in blue-red color scale and significance of correlation is shown on a black-green scale.

(f) Scatter plots of pair-wise comparisons of inclusion level differences of all detected exon junctions in the sALS vs. Ctrl (purple) and fALS vs. Ctrl (pink) datasets generated in this study, and the sALS vs. Ctrl (orange) and C9-ALS vs. Ctrl (green) datasets from the AnswerALS consortium. Events that are called significant in both analyses are depicted in red.

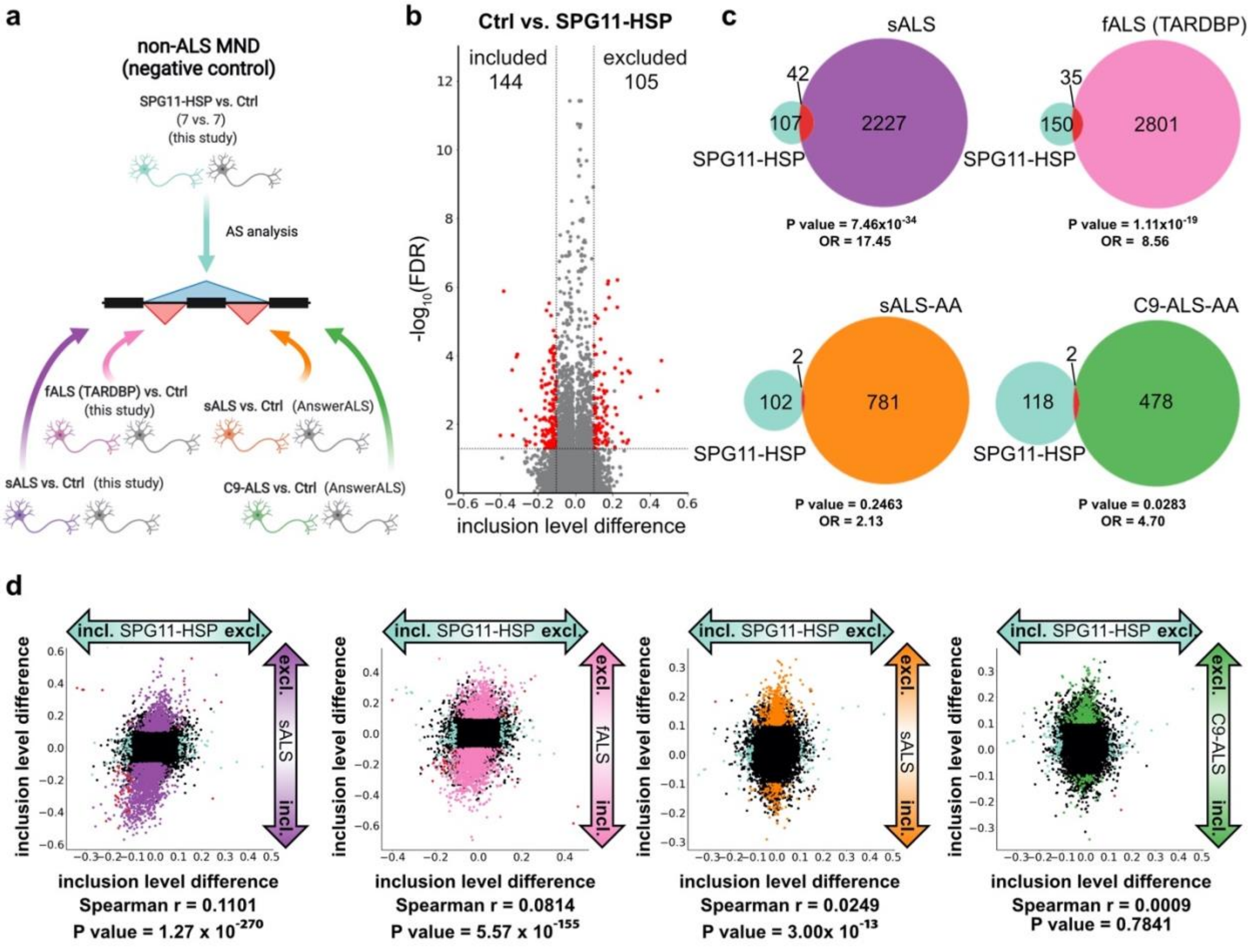

Online Resource Fig. 4. Related to Fig. 2. Degree of AS in SPG11-HSP iPSC-MN

(a) Schematic illustrating the analysis of the SPG11-HSP dataset (n=7 SPG11-HSP; n=7 Ctrl; included is one isogenic pair; cyan) and comparison to the ALS datasets.

(b) Volcano plot showing inclusion level difference (x) and significance (y, negative $\log_{10}$ (FDR)) of AS events significantly differentially spliced (red dots) in SPG11-HSP patients.

(c) Venn diagrams of pairwise comparisons and overlap of significantly differentially alternatively spliced cassette exons between SPG11-HSP and ALS datasets. Significance of overlap was calculated with Fisher's exact test using all events detected in both analyses as background. OR = odds ratio.

(d) Scatter plots of pair-wise comparisons of inclusion level differences of all detected exon junctions in SPG11-HSP (cyan) and sALS vs. Ctrl (purple) and fALS vs. Ctrl (pink) datasets, and the sALS vs. Ctrl (orange) and C9-ALS vs. Ctrl (green) datasets from the

AnswerALS consortium. Events that are called significant in both analyses are depicted in red.

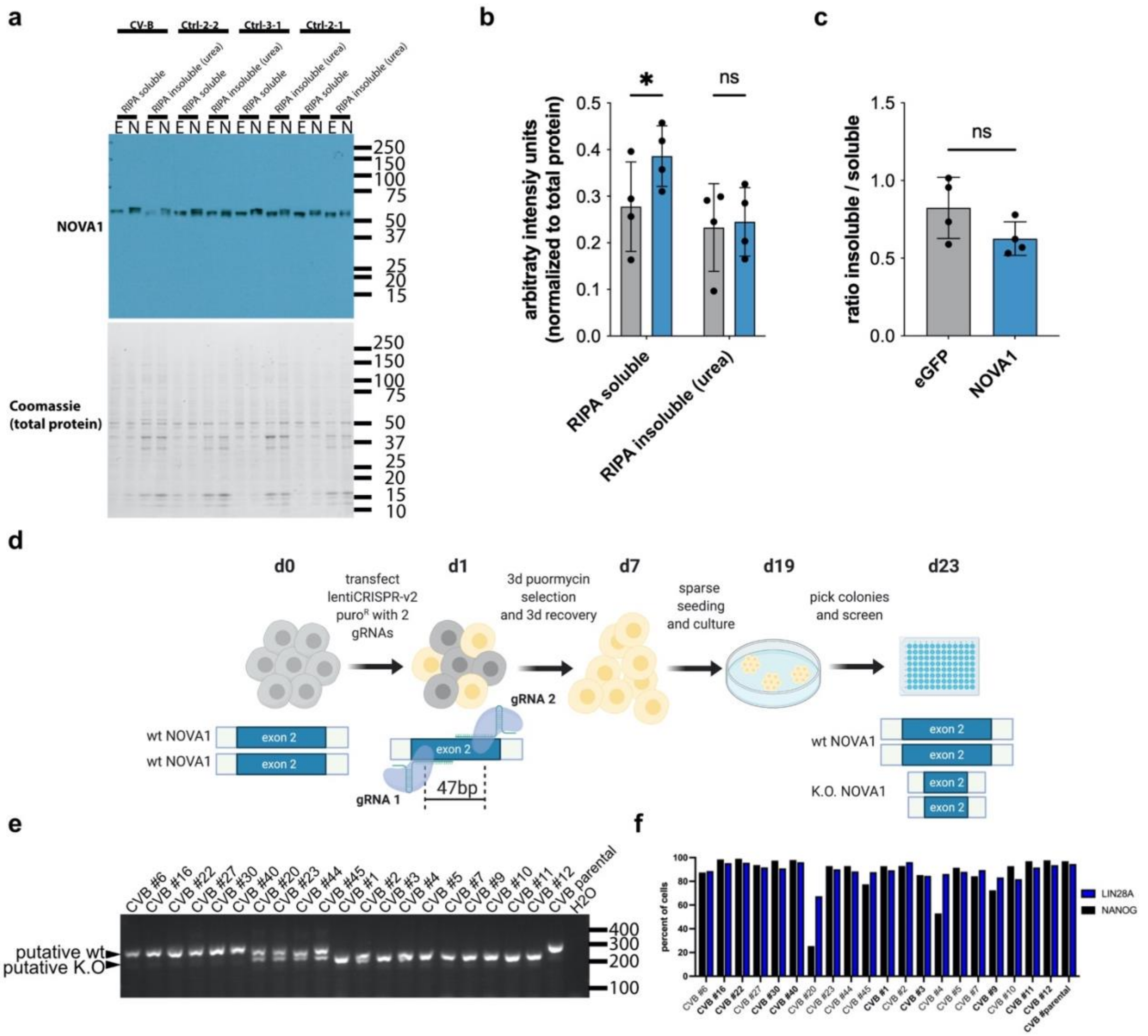

Online Resource Fig. 5. Related to Fig. 4. NOVA1 insolubility upon overexpression and NOVA1 KO iPSC generation

(a) Western blot of NOVA1 in soluble and insoluble fractions and corresponding Coomassie-stained gel (n=4 eGFP overexpressing iPSC-MN (E) and n=4 NOVA1 overexpressing iPSC-MN (N)).

(b) Densitometric quantification of NOVA1 western blot normalized to the Coomassie controls. Statistical significance was determined by 2-way ANOVA. P values were corrected using FDR. Data presented as median ±IQR (gray bars: eGFP overexpression; blue bars: NOVA1 overexpression) and individual values (dots) (n=4 eGFP, n=4 NOVA1). * P value < 0.05; ** P value < 0.01; *** P value < 0.001; **** P value < 0.0001.

(c) Ratio insoluble / soluble of densitometric quantification of western blots normalized to the Coomassie controls. Statistical significance was determined by Mann-Whitney U test. Data presented as median ±IQR (gray bar: eGFP overexpression; blue bar: NOVA1 overexpression) and individual values (dots) (n=4 eGFP, n=4 NOVA1). * P value < 0.05; ** P value < 0.01; *** P value < 0.001; **** P value < 0.0001.

(d) Schematic illustrating methodology for generating NOVA1 K.O. iPSC.

(e) Genotyping of individual clones from NOVA1 KO iPSC generation.

(f) Bar graph of the percentage of NOVA1 iPSC lines generated with NANOG (black) and LIN28A (blue) expression determined via FACS. Cell lines in bold were used for further analyses.

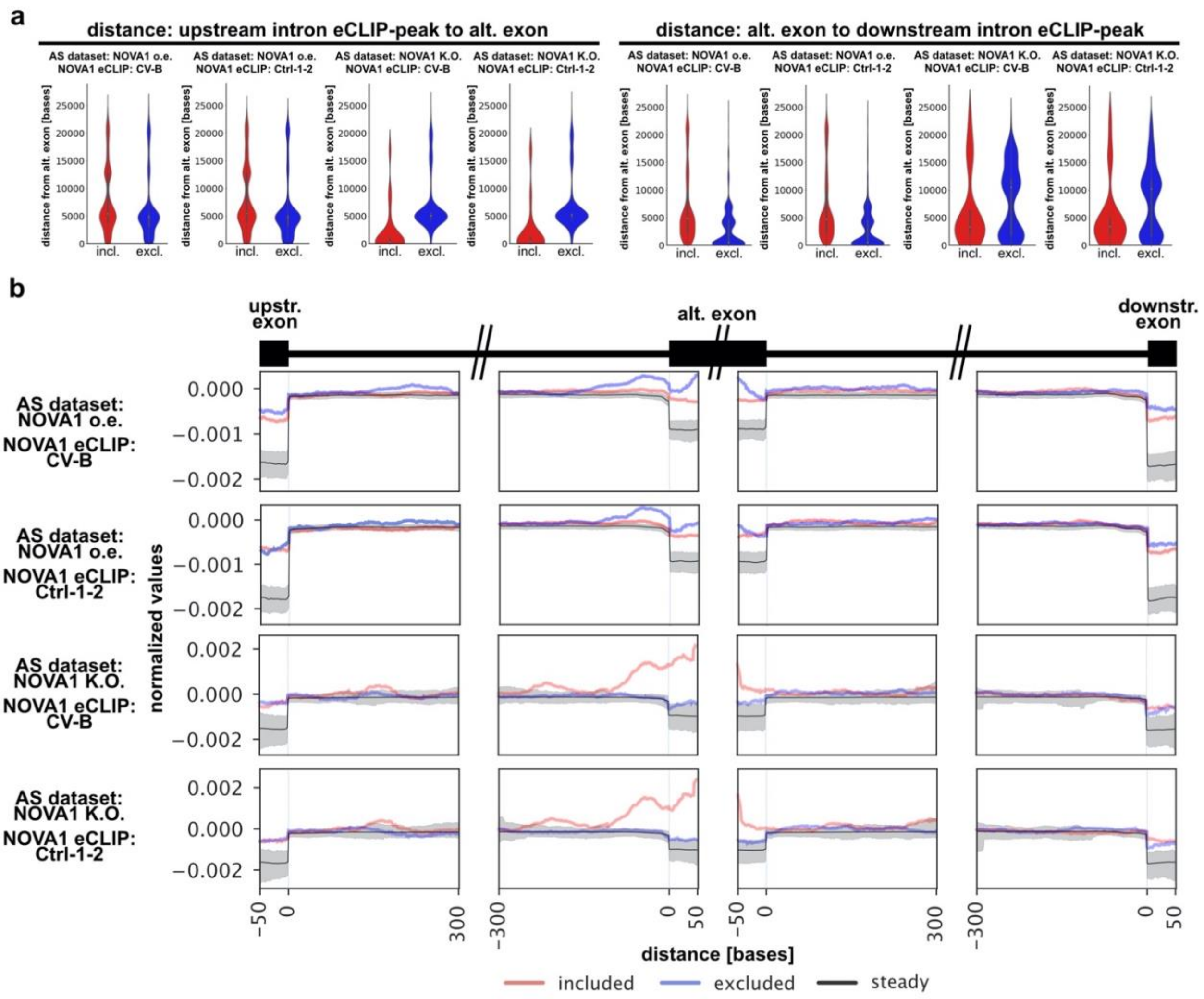

Online Resource Fig. 6. Related to Fig. 4. NOVA1 binding at AS events changed upon altered NOVA1 levels.

(a) Violin plots of the distribution of distances of eCLIP-seq peaks in upstream intron (left graphs, red) and downstream introns (right graphs, blue) to alternative exons in AS events included (red) and excluded (blue) in datasets from altered NOVA1 levels.

(b) RBP binding maps from NOVA1 eCLIP-seq data illustrating NOVA1 binding densities of NOVA1-bound AS events significantly excluded (blue), included (red) or unaltered (black) in a given dataset with altered NOVA1 levels. X axes represent distance in bases.

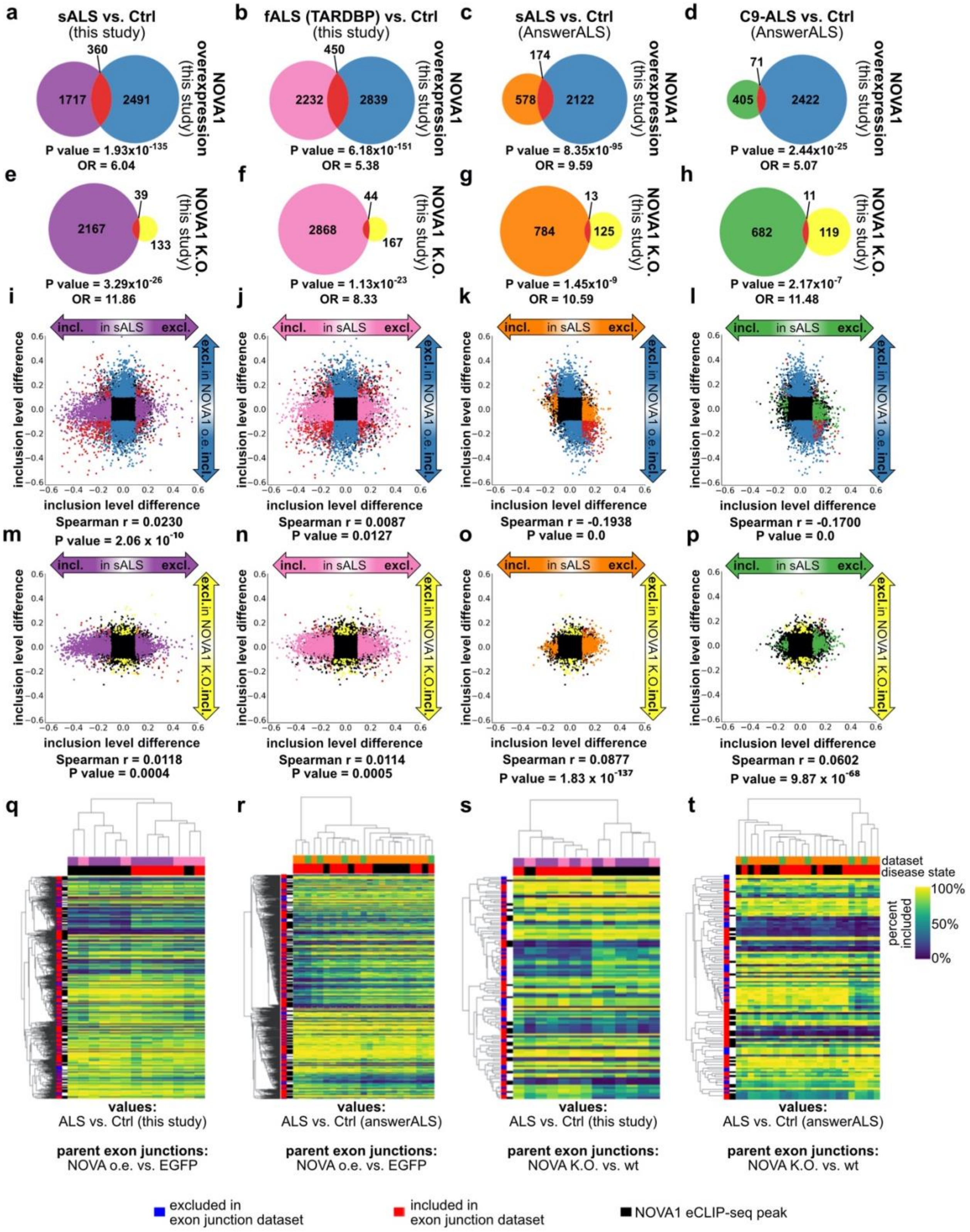

Online Resource Fig. 7. Related to Fig. 5. Inclusion values in ALS and Ctrl samples of AS events altered upon modulation of NOVA1 levels.

(a-d) Venn diagrams of significant differentially alternatively spliced cassette exons between NOVA1 vs. EGFP overexpression (blue) and (a) sALS vs. Ctrl datasets from this study (purple), (b) fALS vs. Ctrl datasets from this study (pink), (c) sALS vs. Ctrl datasets from AnswerALS (orange), (d) C9-ALS vs. Ctrl datasets from AnswerALS (green). Significance of overlap was calculated with Fisher's exact test using all events detected in both analyses as background. OR = odds ratio.

(e-h) Venn diagrams of significant differentially alternatively spliced cassette exons between NOVA1 wt. NOVA1 K.O. (yellow) and (e) sALS vs. Ctrl datasets (purple), (f) fALS vs. Ctrl datasets (pink), (g) sALS vs. Ctrl datasets from AnswerALS (orange), (h) C9-ALS vs. Ctrl datasets from AnswerALS (green). Significance of overlap was calculated with Fisher's exact test using all events detected in both analyses as background. OR = odds ratio.

(i-l) Scatter plot of inclusion level differences of all detected exon junctions in NOVA1 vs. EGFP overexpression (blue) and (i) sALS vs. Ctrl datasets (purple), (j) fALS vs. Ctrl datasets (pink), (k) sALS vs. Ctrl datasets from AnswerALS (orange), (l) C9-ALS vs. Ctrl datasets from AnswerALS (green). Spearman rank correlation coefficient (r) and significance of correlation (P value) are shown below the blot (calculated with scipy.stats.spearmanr). Events that are called significant in both analyses are depicted in red.

(m-p) Scatter plots of inclusion level differences of all detected exon junctions in NOVA1 wt vs. NOVA1 K.O. (blue) and (m) sALS vs. Ctrl datasets (purple), (n) fALS vs. Ctrl datasets (pink), (o) sALS vs. Ctrl datasets from AnswerALS (orange), (p) C9-ALS vs. Ctrl datasets from AnswerALS (green). Spearman rank correlation coefficient (r) significance of correlation (P value) are depicted below the blot (calculated with scipy.stats.spearmanr). Events that are called significant in both analyses are depicted in red.

(q-t) Heatmap of percent spliced-in values of (q) ALS vs. Ctrl from this study in AS events changed in NOVA1 vs. EGFP overexpression, (r) ALS vs. Ctrl from the AnswerALS consortium in AS events changed in NOVA1 vs. EGFP overexpression, (s) ALS vs. Ctrl from this study in AS events changed in NOVA1 wt vs. NOVA1 KO, and (t) ALS vs. Ctrl from the AnswerALS consortium in AS events changed in NOVA1 wt vs. NOVA1 KO.

Clustering was performed using correlation distance. The left columns next to the heatmaps indicate events bound by at least one NOVA1 eCLIP-seq experiment (black: bound, white: not bound). The right columns next to the heatmaps indicate AS inclusion state in datasets from altered NOVA1 expression (red: included, blue: excluded). The top bars above the heatmaps indicate the dataset origin of a given sample (purple: sALS vs. Ctrl, this study; pink: fALS vs. Ctrl, this study; orange: sALS vs. Ctrl, AnswerALS; and green: C9-ALS vs. Ctrl, AnswerALS). The bottom bar above the heatmap represent disease state of a sample (black: Ctrl); red: ALS).

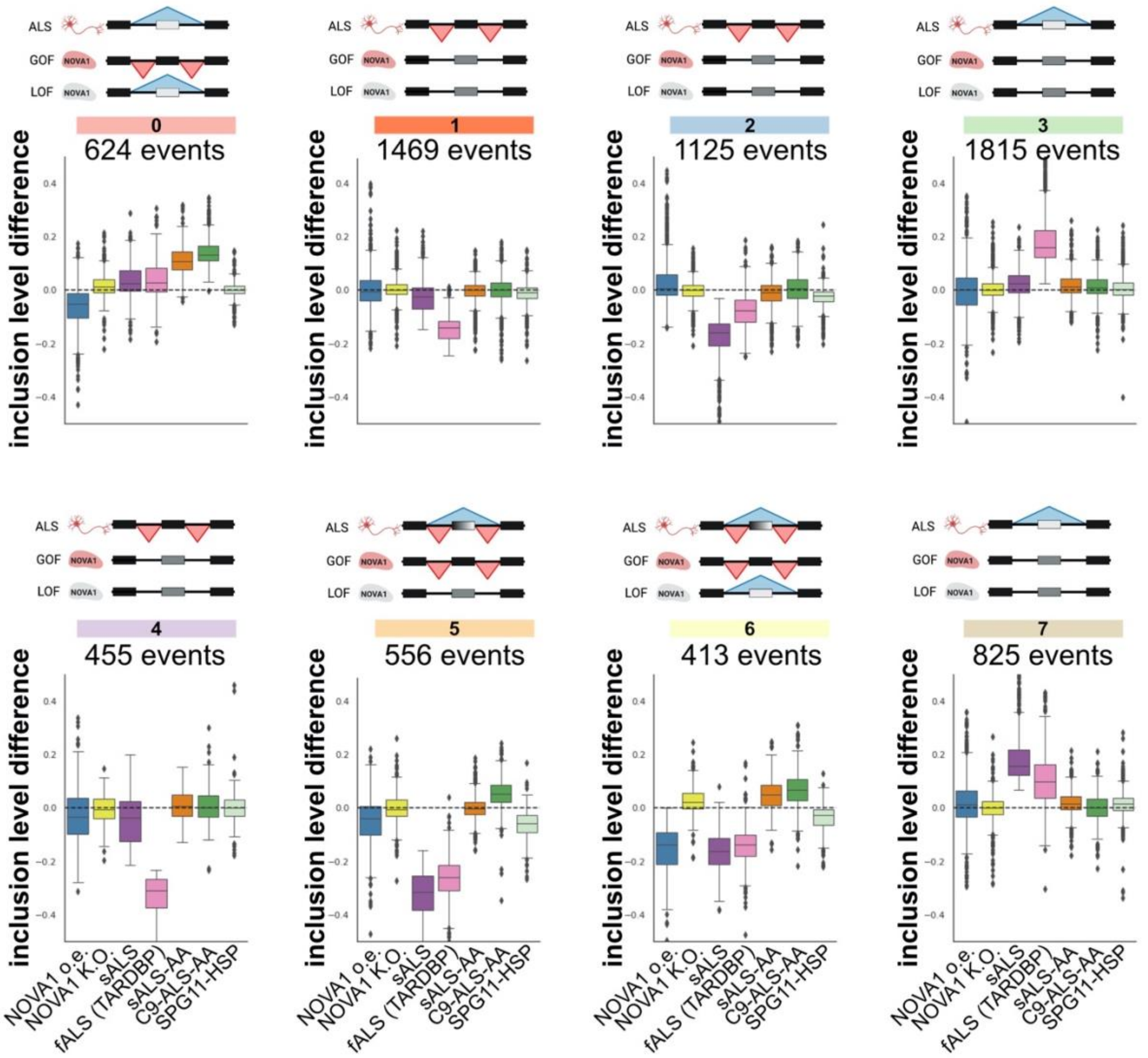

Online Resource Fig. 8. Related to Fig. 5. Individual AS clusters in ALS and SPG11-HSP.

Boxplots of inclusion level differences in clusters 0 - 7 (sALS vs. Ctrl: purple; fALS vs. Ctrl: pink; sALS vs. Ctrl (AA): orange; C9-ALS vs. Ctrl (AA): green; SPG11-HSP vs. Ctrl: cyan; NOVA1 vs. EGFP overexpression (blue) and NOVA1 wt vs. NOVA1 K.O. (yellow). Ideogram on top illustrate the direction of the most prominent observed change in ALS, NOVA1 gain of function (GOF) and NOVA1 loss of function (LOF) (from top to bottom).

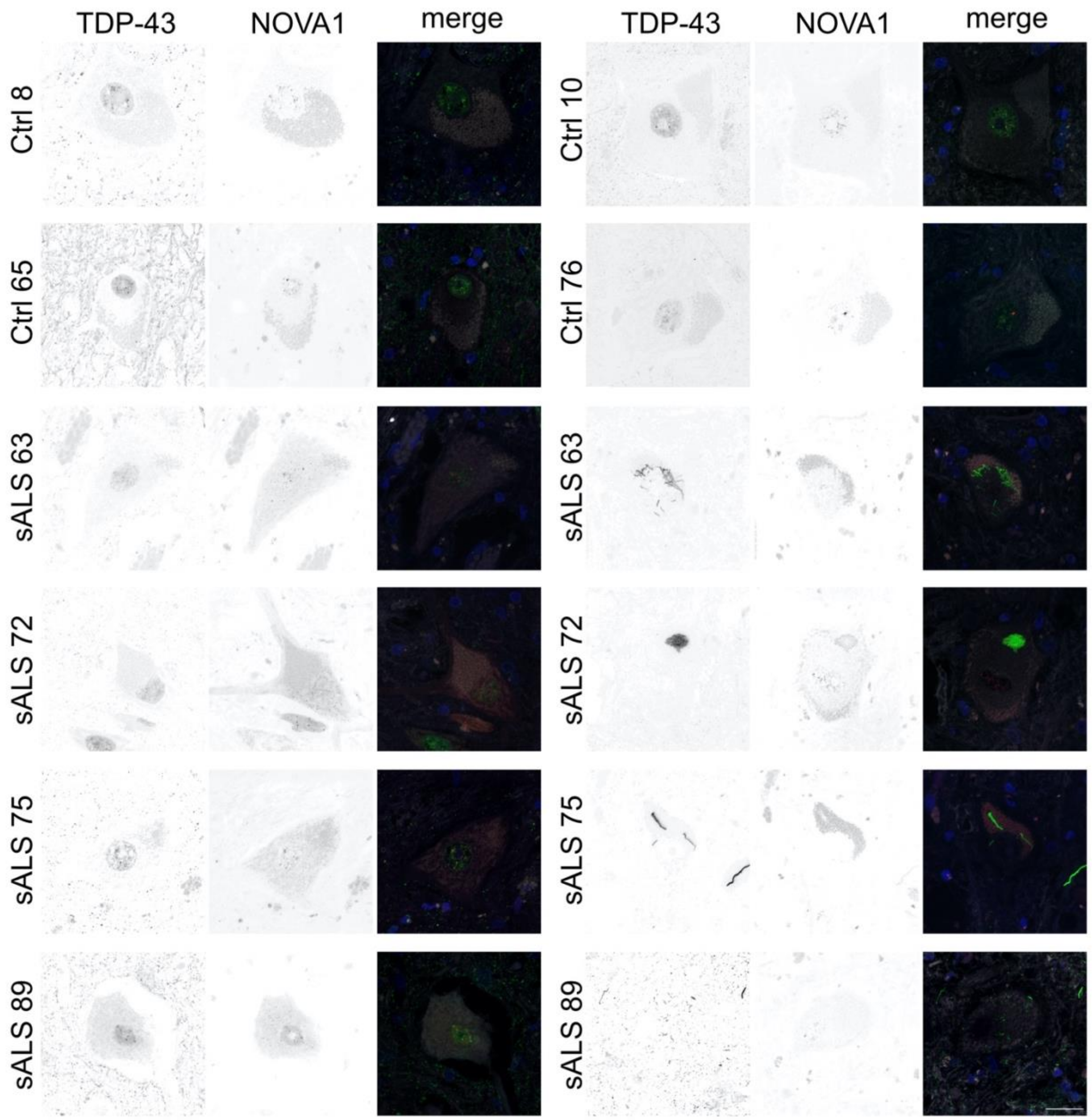

Online Resource Fig. 9. Related to Fig. 6. Spectrum of TDP-43 pathology and corresponding NOVA1 staining postmortem spinal cord tissue of sALS.

Representative images of MN in sALS patient postmortem tissue. Pictures stained for TDP-43 (green), NOVA1 (red), bIII-tubulin (gray) and DAPI (blue). Scale bars: 25µm.

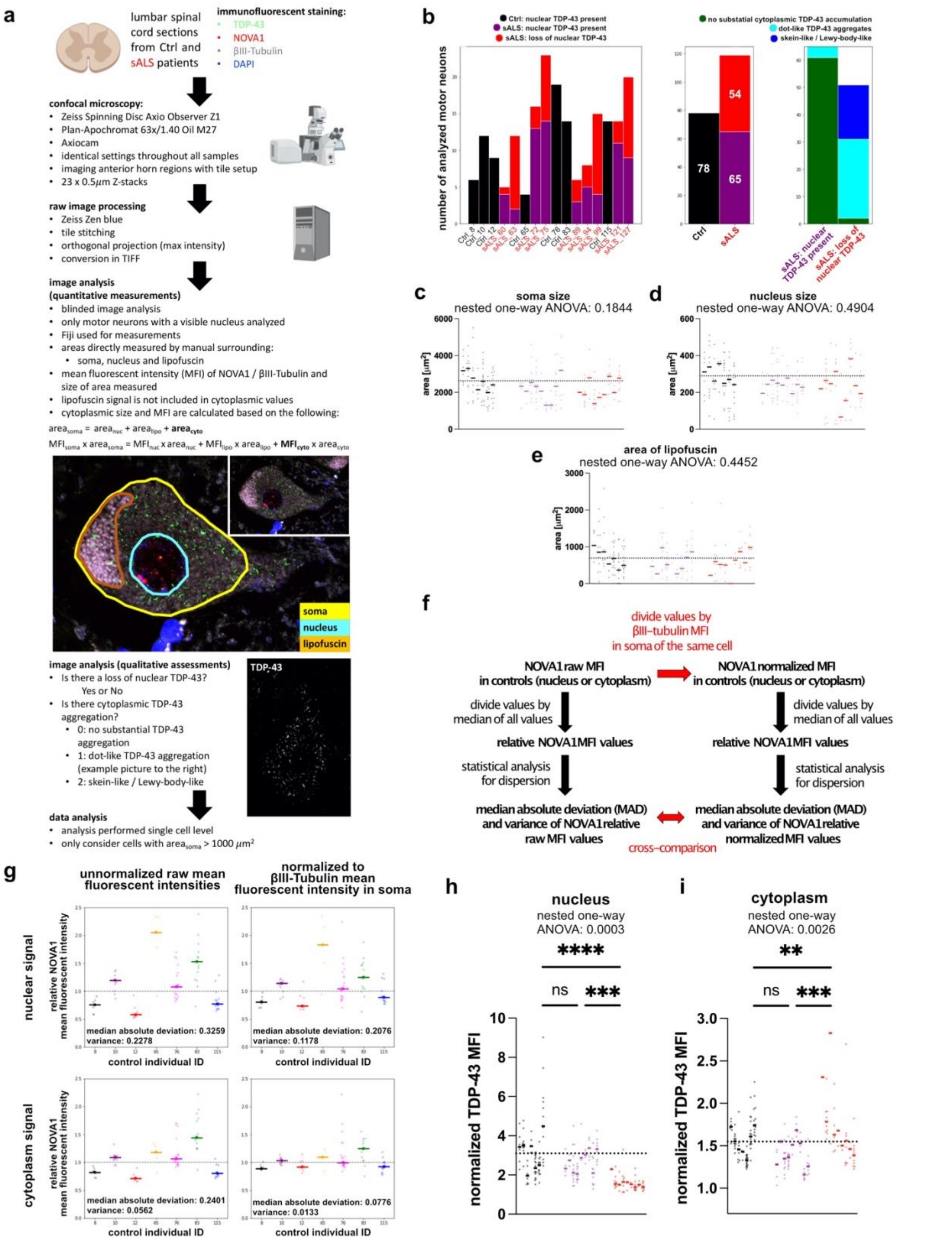

Online Resource Fig. 10. Related to Fig. 6. Analysis of human spinal cord samples from ALS patients and controls.

(a) Schematic illustrating detailed analysis strategy and pipeline for tissue section analysis.

(b) Bar graphs of the number of analyzed MNs and their respective status regarding TDP-43 pathology (purple: retained nuclear TDP-43, red: loss of nuclear TDP-43, green: no cytoplasmic TDP-43 accumulation, cyan: dot-like cytoplasmic TDP-43 aggregation, blue: skein-like/Lewy-body-like TDP-43 aggregation, black: Ctrl.

(c-e) Scatter plots illustrating (c) soma size, (d) nucleus size and (e) lipofuscin area of individual MNs (dots) in the different individuals analyzed (line) Ctrl (black), sALS with nuclear TDP-43 (purple) and sALS with loss of nuclear TDP-43 (red). Dashed horizontal line represent median in Ctrl. Statistical differences between the three groups were determined by nested one-way ANOVA.

(f) Schematic illustrating bIII-tubulin normalization strategy to NOVA1 intensities.

(g) Scatter plots of relative nuclear (top) and cytoplasmic (bottom) NOVA1 staining intensity of non-normalized (left) and bIII-tubulin normalized (right) values. Horizontal lines indicate medians. Statistical analysis performed on medians from individuals.

(h) Scatter plot of bIII-tubulin normalized mean nuclear TDP-43 staining intensities of single MNs (dots) their average in an individual (line). Dashed represents median in Ctrl. Statistical significance was determined by nested one-way ANOVA. * P value < 0.05; ** P value < 0.01; *** P value < 0.001; **** P value < 0.0001. Significant variation between individuals within a group: P value < 0.0001.

(i) Scatter plot of bIII-tubulin normalized mean cytoplasmic TDP-43 intensities of single MN (dots) their average in an individual (line). Dashed represents median in Ctrl. Statistical significance was determined by nested one-way ANOVA. * P value < 0.05; ** P value < 0.01; *** P value < 0.001; **** P value < 0.0001. Significant variation between individuals within a group: P value > 0.05.

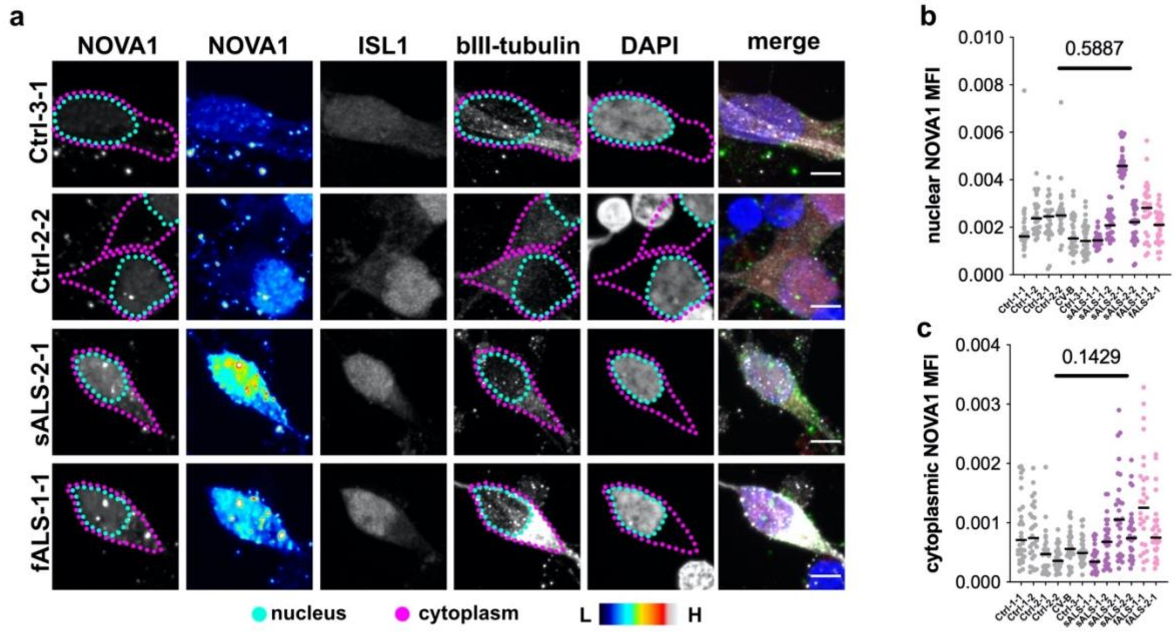

Online Resource Fig. 11. Related to Figure 6. NOVA1 localization in Ctrl and ALS iPSC-MN

(a) Representative images of iPSC-MNs stained for NOVA1 (green), ISL1 (red), bIII-tubulin (gray) and DAPI (blue). Analyzed areas are indicated by the dashed lines (nuclear area, cyan; cytoplasmic area, fuchsia). NOVA1 fluorescence intensities are also illustrated in an intensity heatmap. Scale bar: 5µm.

(b) Scatter plot of nuclear NOVA1 mean fluorescent intensities (MFI) of individual ISL1 MN (n=28/cell line; dots) in Ctrl (n=6) and ALS (n=6). Black bar represents median in a cell line. Statistical significance was determined on medians by Mann-Whitney U test. P value = 0.5887.

(c) Scatter plot of cytoplasmic NOVA1 mean fluorescent intensities (MFI) of individual ISL1 MN (n=28/cell line; dots) in Ctrl (n=6) and ALS (n=6). Black bar represents median in a cell line. Statistical significance was determined on medians by Mann-Whitney U test. P value = 0.1429.

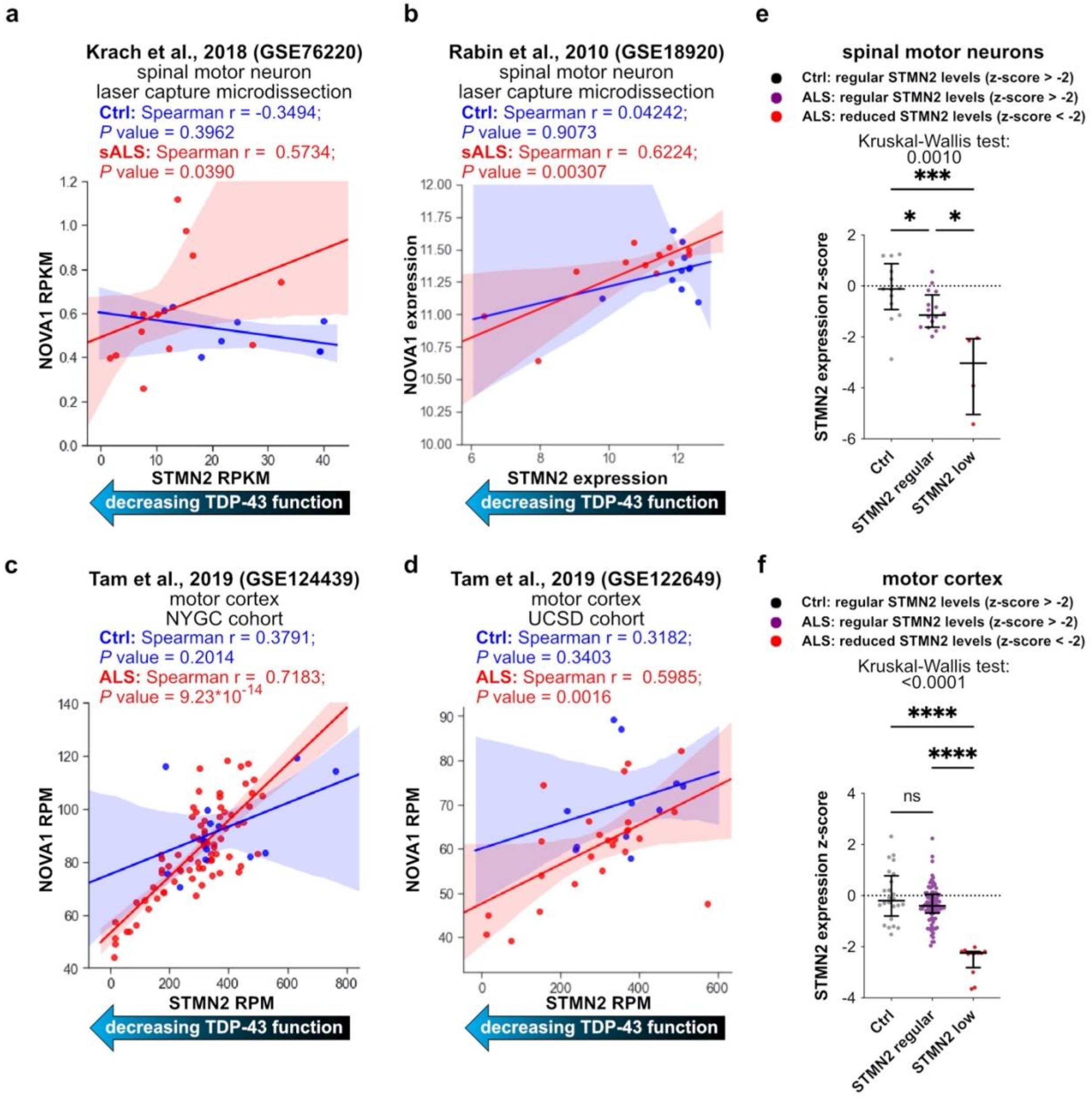

Online Resource Fig. 12. Related to Fig. 6. Correlation of NOVA1 and STMN2 expression values in publicly available datasets.

(a-d) Scatter plot of the correlation of NOVA1 and STMN2 expression in ALS (red) and Ctrl (blue) samples in publicly available datasets from (a) RNA-seq from LCM MNs [32], (b) microarray from LCM MNs [55], (c) RNA-seq from motor cortex from NYGC and (d) the UCSD cohort [63]. Spearman rank correlation coefficients in Ctrl and ALS and significance of correlation are shown above each graph.

(e) Scatter plot of STMN2 expression Z-scores from LCM MN from [32] and [55] based on Ctrl STMN2 expression in a dataset. Medians and interquartile ranges are shown. Statistical significance was determined by Kruskal-Wallis test and Dunn's post hoc test. * P value < 0.05; ** P value < 0.01; *** P value < 0.001; **** P value < 0.0001.

(f) Scatter plot of STMN2 expression Z-scores from motor cortex datasets from NYGC and the UCSD cohort [63] based on Ctrl STMN2 expression in a dataset. Medians and interquartile ranges are shown.. Statistical significance was determined by Kruskal-Wallis test and Dunn's post hoc test. * P value < 0.05; ** P value < 0.01; *** P value < 0.001; **** P value < 0.0001.

patient	iPSC-line	sex	diagnosis	family history	pathogenic variant	age at onset	age at biopsy	UMN signs	LMN signs	line ID	origin	repro-gramming
Ctrl-1	Ctrl-1-1	M	Ctrl	no	no	N/A	26	N/A	N/A	UKERiff2-X-18	UKER	RV
	Ctrl-1-2									UKERiff2-SC-11	UKER	SV
Ctrl-2	Ctrl-2-1	F	Ctrl	no	no	N/A	45	N/A	N/A	UKERi33q-E-2	UKER	RV
	Ctrl-2-2									UKERi33q-E-6	UKER	RV
Ctrl-3	Ctrl-3-1	F	Ctrl	yes*	no	N/A	52	N/A	N/A	KinI-ALS-17.2	UCSD	SV
	Ctrl-3-2									KinI-ALS-17.5	UCSD	SV
CV	CV-B	M	Ctrl	no	no	N/A	N/A	N/A	N/A	CV-B	Gore et al.	RV
sALS-1	sALS-1-1	M	ALS	no	no	18	22	yes	yes	UKERiu5q-E-8	UKER	RV
	sALS-1-2									UKERiu5q-SC-1	UKER	SV
sALS-2	sALS-2-1	M	ALS	no	no	47	50	yes	yes	UKERizxx-E-10	UKER	RV
	sALS-2-2									UKERizxx-E-16	UKER	RV
fALS-1	fALS-1-1	M	ALS	yes	TDP-43^{N352S}	N/A	53	N/A	N/A	ALS17.5	UCSD	SV
fALS-2	fALS-2-1	F	ALS	yes	TDP-43^{G298S}	N/A	N/A	N/A	N/A	t.Bird2	Alami et al.	RV

Online Resource Table 1. Related to Figure 1. Patient specifications for iPSC used in this study.

F = female, M = Male, UCSD = University of California San Diego, UKER = Universitätsklinikum Erlangen, RV = retrovirus, SV = Sendai virus, * Ctrl-3 and fALS-1 are siblings. CV in CV-B stands for the donor Craig Venter.

patient_ID	assigned_group
CASE-NEUEM720BUU	C9-ALS
CASE-NEUTN952DDG	C9-ALS
CASE-NEUYY225MNZ	C9-ALS
CASE-NEUCE965ZGK	C9-ALS
CASE-NEUDT709YHN	C9-ALS
CTRL-NEUAJ025JC3	Ctrl
CTRL-NEUCV809LL4	Ctrl
CTRL-NEUDM126GNG	Ctrl
CTRL-NEUEY565NWT	Ctrl
CTRL-NEUFZ500KDB	Ctrl
CTRL-NEUFZ508VBV	Ctrl
CTRL-NEUJX341NDP	Ctrl
CTRL-NEUKW131XJ2	Ctrl
CTRL-NEURJ861MMD	Ctrl
CASE-NEUAF553MJ3	sALS

CASE-NEUAM655HF7	sALS
CASE-NEUDG000ZG5	sALS
CASE-NEUGW326BRV	sALS
CASE-NEUPN525XEW	sALS
CASE-NEUTA689LN5	sALS
CASE-NEUVM674HUA	sALS
CASE-NEUAG603XLK	sALS
CASE-NEUVF888UHM	sALS

Online Resource Table 2. Related to Figure 2. AnswerALS datasets used for final analysis.

patient number	primary diagnosis	age	gender	disease onset	disease duration
10	Control	78	Male	N/A	N/A
76	Control	68	Male	N/A	N/A
115	Control	94	Male	N/A	N/A
65	Control	82	Male	N/A	N/A
83	Control	63	Female	N/A	N/A
8	Control (AD)	N/A	N/A	N/A	N/A
12	Control (AD)	N/A	N/A	N/A	N/A
60	SALS	58	Female	Bulbar	3
63	SALS	68	Male	Arm	2.5
72	SALS	77	Female	Bulbar	6
75	SALS	52	Male	Arm	3.5
89	SALS	36	Male	Bulbar	3
94	SALS	62	Male	Bulbar	7
99	SALS	70	Female	Bulbar	2.5
121	SALS	67	Male	Right hand	2.5
127	SALS	67	Male	Right hand/Respiratory	1.5

Online Resource Table 3. Related to Figure 6. Summary of individuals used for postmortem imaging analysis. AD = Alzheimer’s disease

REAGENT or RESOURCE	SOURCE	IDENTIFIER
Antibodies		
rabbit anti-TDP-43	Bethyl Laboratories Inc.	A303-223A
mouse anti-TDP-43	Novus Biologicals	H00023435-M01
rabbit anti-NOVA1	abcam	ab183024
rabbit anti-NOVA2	ProteinTech	55002-1-AP
rabbit anti-beta-III-Tubulin	abcam	ab18207
chicken anti-beta-III-Tubulin	Millipore	AB9354
chicken anti-beta-III-Tubulin	abcam	ab107216
rabbit anti-RBM9 (RBFOX2)	Bethyl Laboratories Inc.	A300-864A
rabbit anti-RBFOX3	abcam	ab177487
mouse anti-ELAVL4	Santa Cruz Biotechnology	sc-48421
mouse anti-FXR2	ThermoFisher	MA1-16767
mouse anti-ISL1	DSHB	39.4D5-s
rabbit anti-OLIG2	Millipore	AB9610
mouse anti-GAPDH	Millipore	CB1001
anti-PAX6-APC	Miltenyi Biotec	130-123-267
anti-NESTIN-PerCp-Cy5.5	BD Biosciences	561231

anti-beta-III-Tubulin-PB	novusbio	NB600-1018AF405
anti-ISL1-PE	BD Biosciences	562547
anti-rabbit-IgG-488	ThermoFisher	A21449
anti-rabbit-IgG-546	ThermoFisher	A10040
anti-mouse-IgG-546	ThermoFisher	A10036
anti-chicken-IgY 647	Santa Cruz Biotechnology	sc-2718
anti-rabbit-IgG-HRP	ThermoFisher	SA1-200
anti-mouse-IgG-HRP	ThermoFisher	SA1-100
Bacterial and virus strains		
One Shot™ Stbl3™ Chemically Competent E. coli	ThermoFisher	C737303
Biological samples		
Human spinal cord tissue sections	John Ravits, University of California, San Diego	N/A
Chemicals, peptides, and recombinant proteins		
Matrigel	Corning	354230
Geltrex	ThermoFisher	A1413302
Poly-D-Lysine	Sigma-Aldrich	P6407-5mg
Laminin	ThermoFisher	23017-015
Laminin	Sigma-Aldrich	L2020

mTeSR Plus 5X Supplement	STEMCELL Technologies	100-0275/100ml
mTESR Plus Basal Medium	STEMCELL Technologies	100-0274
DMEM/F12+Glutamax	ThermoFisher	31331028
Pen/Strep	ThermoFisher	15140122
N2 Supplement	Invitrogen	17502-048
B27 Supplement	Invitrogen	17504-044
Y-27632 (ROCK inhibitor)	Tocris	1254
Dorsomorphin	Tocris	3093
SB431542	Miltenyi Biotec	130-106-543
CHIR99021	Tocris	4423
Retinoic acid	Tocris	1254
SAG	Tocris	4366
DAPT	Tocris	2634
BDNF	PeproTech	450-02
GDNF	PeproTech	450-10
CNTF	PeproTech	450-13
Sodium arsenate	Sigma Aldrich	S7400
ReLeSR	STEMCELL Technologies	05872

Accutase	ThermoFisher	A11105-01
Triple	Gibco	12605-010
FBS	ThermoFisher	10500-064
OptiMEM	ThermoFisher	51985026
Puromycin	Gibco	A11138-03
CloneR	Stem Cell Technologies	05888
Critical commercial assays		
Lipofectamine 3000	ThermoFisher	L3000008
660-DEVD-FMK caspase-3/7 inhibitor reagent	abcam	ab270785
Live-or-Dye	Biotium	32008-T
Deposited data		
Uncropped western blots	This paper	http://dx.doi.org/10.17632/v7p6dh5tvc.1
FACS raw data (fcs files)	This paper	http://dx.doi.org/10.17632/v7p6dh5tvc.1
MaxQuant quantification of peptides from mass spectrometry	This paper	http://dx.doi.org/10.17632/v7p6dh5tvc.1
rMATS outputs	This paper	http://dx.doi.org/10.17632/v7p6dh5tvc.1
counts files	This paper	http://dx.doi.org/10.17632/v7p6dh5tvc.1
DESeq2 outputs	This paper	http://dx.doi.org/10.17632/v7p6dh5tvc.1
eCLIP-seq peaks (bed file, unfiltered)	This paper	http://dx.doi.org/10.17632/v7p6dh5tvc.1

Postmortem image analysis	This paper	http://dx.doi.org/10.17632/v7p6dh5tvc.1
RNA-seq: sALS vs. Ctrl	This paper	EGAD00001008424, EGA, ega-archive.org
RNA-seq: fALS vs. Ctrl	This paper	EGAD00001008425, EGA, ega-archive.org
eCLIP-seq: TDP-43	This paper	EGAD00001008426, EGA, ega-archive.org
eCLIP-seq: NOVA1, NOVA2, RBFOX2	This paper	EGAD00001008428, EGA, ega-archive.org
RNA-seq: EGFP vs. NOVA1 overexpression	This paper	EGAD00001008423, EGA, ega-archive.org
RNA-seq: NOVA1 wt vs. NOVA1 K.O.	This paper	EGAD00001008427, EGA, ega-archive.org
Experimental models: Cell lines		
iPSC: CV-B	[24]	N/A
iPSC: KinIALS17.5 (Ctrl-3-1)	[32]	N/A
iPSC: ALS17.5 (fALS-1-1)	[32]	N/A
iPSC: t.Bird2 (fALS-2-1)	[2]	N/A
iPSC: UKERiff2-X-18 (Ctrl-1-1)	This paper	N/A
iPSC: UKERiff2-SC-11 (Ctrl-1-2)	This paper	N/A
iPSC: UKERi33q-E-2 (Ctrl-2-1)	[27]	N/A
iPSC: UKERi33q-E-6 (Ctrl-2-2)	[27]	N/A
iPSC: UKERiu5q-E-8 (sALS-1-1)	This paper	N/A
iPSC: UKERiu5q-SC-1 (sALS-1-2)	This paper	N/A

iPSC: UKERizxx-E-10 (sALS-2-1)	This paper	N/A
iPSC: UKERizxx-E-16 (sALS-2-2)	This paper	N/A
iPSC: CV-B NOVA1 K.O. #1	This paper	N/A
iPSC: CV-B NOVA1 K.O. #3	This paper	N/A
iPSC: CV-B NOVA1 K.O. #9	This paper	N/A
iPSC: CV-B NOVA1 K.O. #11	This paper	N/A
iPSC: CV-B NOVA1 K.O. #12	This paper	N/A
iPSC: CV-B NOVA1 wt. #16	This paper	N/A
iPSC: CV-B NOVA1 wt. #22	This paper	N/A
iPSC: CV-B NOVA1 wt. #30	This paper	N/A
iPSC: CV-B NOVA1 wt. #40	This paper	N/A
iPSC: UKERi4AA-S006	[25]	N/A
iPSC: UKERi4AA-S-14A	[25]	N/A
iPSC: UKERiK22-S-001	[25]	N/A
iPSC: UKERiK22-S-003	[25]	N/A
iPSC: UKERiG7G-S-001	[53]	N/A
iPSC: UKERiG7G-S-008	[53]	N/A
iPSC: UKERi33Q-S-006	[25]	N/A

iPSC: UKERi33Q-R-106	[25]	N/A
iPSC: UKERi82A-S-004	[25]	N/A
iPSC: UKERi82A-S022	[25]	N/A
iPSC: UKERi55O-S-002	[25]	N/A
iPSC: UKERi55O-S-004	[25]	N/A
ES: HuES6 clone 2, (wt)	[53]	N/A
ES: HuES6 clone 15 (SPG11 K.O.)	[53]	N/A
Human HEK293T		
Recombinant DNA		
Plasmid: Lenti-CRISPR v2	[57]	Addgene plasmid #52961
Plasmid: LV-CAG-EGFP-P2A-PuroR	This paper	N/A
Plasmid: LV-CAG-NOVA1-P2A-PuroR	This paper	N/A
Software and algorithms		
STAR	[18]	https://github.com/alexdobin/STAR
Cutadapt	[43]	https://cutadapt.readthedocs.io/en/stable/
Samtools	[34]	http://samtools.sourceforge.net/
Python	Python Software Foundation	https://www.python.org/
GraphPad Prism 9	GraphPad Software, Inc.	http://graphpad.com/scientific-software/prism

Pybedtools	[14]	https://daler.github.io/pybedtools/
Subread	[35]	http://subread.sourceforge.net/
ZEN blue	Zeiss	https://www.zeiss.com/microscopy/us/products/microscope-software.html
rMATS	[60]	http://rnaseq-mats.sourceforge.net/
Fiji	[58]	https://imagej.net/Fiji
CytExpert	Beckman Coulter	https://www.beckman.com/flow-cytometry/instruments/cytoflex/software
CLIPper	[39]	https://github.com/YeoLab/clipper
CellProfiler	[45]	https://cellprofiler.org/
Scikit-learn	[51]	https://scikit-learn.org/
Other		
AnswerALS RNA-seq datasets	AnswerALS consortium	http://data.answerals.org
Laser capture microdissection RNA-seq dataset form ALS patients and Ctrl	[32]	GSE76220
Laser capture microdissection microarray dataset form ALS patients and Ctrl	[55]	GSE18920
Motor cortex RNA-seq datasets from ALS patients and Ctrl	[63]	GSE122649<br>GSE124439
iPSC-MN, TARDBP knock down	[31]	GSE121569
SH-SY5Y, TARDBP knock down	[46]	GSE122069
Mouse striatum, TARDBP knock down	[52]	GSE27394
Mouse brain, TDP-43-dNLS	[3]	GSE65973

iPSC-MN, puromycin stressed	[41]	GSE157467

Online Resource Table 4. Detailed list of materials, biological samples, software and data used in this study.
